# Association between antibody responses post-vaccination and severe COVID-19 outcomes in Scotland

**DOI:** 10.1038/s41541-024-00898-w

**Published:** 2024-06-14

**Authors:** Calum Macdonald, Norah Palmateer, Andrew McAuley, Laura Lindsay, Taimoor Hasan, Safraj Shahul Hameed, Elliot Hall, Karen Jeffrey, Zoë Grange, Petros Gousias, Sally Mavin, Lisa Jarvis, J. Claire Cameron, Luke Daines, Holly Tibble, Colin R. Simpson, Colin McCowan, Srinivasa Vittal Katikireddi, Igor Rudan, Adeniyi Francis Fagbamigbe, Lewis Ritchie, Ben Swallow, Paul Moss, Chris Robertson, Aziz Sheikh, Josie Murray

**Affiliations:** 1 Usher Institute, University of Edinburgh, Teviot Pl, EH8 9AG Edinburgh, UK; 2 Health Data Research UK, Gibbs Building, 215 Euston Road, NW1 2BE London, UK; 3 School of Health and Life Sciences, Glasgow Caledonian University, Cowcaddens, Road, Glasgow, G4 0BA UK; 4 Public Health Scotland, Meridian Court, 5 Cadogan Street, G2 6QE Glasgow, UK; 5 Scottish Microbiology Reference Laboratory, Raigmore Hospital, Old Perth Road, Inverness, IV2 3UJ UK; 6 Scottish National Blood Transfusion Service, Jack Copland Centre, 52 Research Avenue North, EH14 4BE Edinburgh, UK; 7 School of Health, Wellington Faculty of Health, Victoria University of Wellington, PO Box 600, Wellington, 6140 Wellington New Zealand; 8 School of Medicine, University of St Andrews, North Haugh, St Andrews, KY16 9TF UK; 9 MRC/CSO Social & Public Health Sciences Unit, University of Glasgow Berkeley Square, 99 Berkeley St., G3 7HR Glasgow, UK; 10 Institute of Applied Health Sciences, University of Aberdeen, Polwarth Building, Foresterhill Rd, AB25 2ZD Aberdeen, UK; 11 Centre of Academic Primary Care, University of Aberdeen, Polwarth Building, Foresterhill Rd, AB25 2ZD Aberdeen, UK; 12 School of Mathematics and Statistics, University of St Andrews, KY16 9SS St Andrews, UK; 13 Institute of Immunology and Immunotherapy, University of Birmingham, Cancer Sciences Building, Edgbaston, B15 2TT Birmingham, UK; 14 Department of Mathematics and Statistics, University of Strathclyde, Richmond Street Glasgow, G1 1XH Glasgow, UK; 15 Nuffield Department of Primary Care Health Sciences, University of Oxford, Oxford, OX2 6GG Oxford, UK

**Keywords:** Risk factors, RNA vaccines

## Abstract

Several population-level studies have described individual clinical risk factors associated with suboptimal antibody responses following COVID-19 vaccination, but none have examined multimorbidity. Others have shown that suboptimal post-vaccination responses offer reduced protection to subsequent SARS-CoV-2 infection; however, the level of protection from COVID-19 hospitalisation/death remains unconfirmed. We use national Scottish datasets to investigate the association between multimorbidity and testing antibody-negative, examining the correlation between antibody levels and subsequent COVID-19 hospitalisation/death among double-vaccinated individuals. We found that individuals with multimorbidity ( ≥ five conditions) were more likely to test antibody-negative post-vaccination and 13.37 [6.05–29.53] times more likely to be hospitalised/die from COVID-19 than individuals without conditions. We also show a dose-dependent association between post-vaccination antibody levels and COVID-19 hospitalisation or death, with those with undetectable antibody levels at a significantly higher risk (HR 9.21 [95% CI 4.63–18.29]) of these serious outcomes compared to those with high antibody levels.

## Introduction

The COVID-19 vaccination programme in Scotland has rolled out up to five doses for some individuals to date (March 10, 2023)^1^ and the vaccines have been shown to be highly protective against severe outcomes of the associated disease^2,3^. However, immunological responses to these vaccines differ between individuals and there may be reduced vaccine clinical effectiveness in those with suboptimal immune responses.

Most individuals generate SARS-CoV-2-specific antibodies following COVID-19 vaccination or natural infection. While neutralising antibodies are widely accepted as markers of protection against future infection^4^, the total SARS-CoV-2-specific antibody titre is more practical to measure and can be deployed in large-scale studies^5^. Previous population-based studies have examined demographic, clinical and lifestyle factors associated with suboptimal post-vaccination IgG responses and observed lower antibody levels in older people, males, transplant recipients, obese individuals, smokers and those with specific comorbidities (e.g., cancer, depression, diabetes, hypertension, immunodeficiency, stroke, and kidney, liver, lung or neurological disease)^6,7^. Additionally, previous studies have shown a correlation between post-vaccination IgG and protection from subsequent SARS-CoV-2 infection^5,8–12^. To our knowledge, however, no studies have examined the association between multimorbidity and post-vaccination immune responses, and, crucially, questions remain as to the level of protection from severe COVID-19 sequelae (i.e., hospitalisation and death) among those with suboptimal post-vaccination immune responses. Answering these questions could illustrate the potential clinical utility of measuring IgG and help to identify individuals who should be targeted for modified vaccination strategies or COVID-19 therapeutics.

The COVID-19 vaccine programme began on 8 December 2020 in Scotland. The first doses of the ChAdOx1 vaccine were administered to care home residents and frontline health and social care workers. The eligibility for vaccination began with individuals aged 80 and over, progressing to younger age groups with a higher priority for those with underlying health conditions. Notably, in February 2021, the BNT162b2 vaccine was introduced and as the rollout progressed, by April 2021, mRNA-1273 was also added to the programme.

Here we use a novel, real-time, national linked dataset to investigate risk factors associated with testing negative for anti-SARs-CoV-2 IgG after completion of the primary COVID-19 vaccine course (at least two doses of ChAdOx1 [Oxford/AstraZeneca], BNT162b2 [Pfizer BioNTech] and/or mRNA-1273 [Moderna]). We also investigate the association between post-vaccination IgG levels and the risk of COVID-19 infection and hospitalisation or death.

## Results

### Participants

**Fig. 1:**
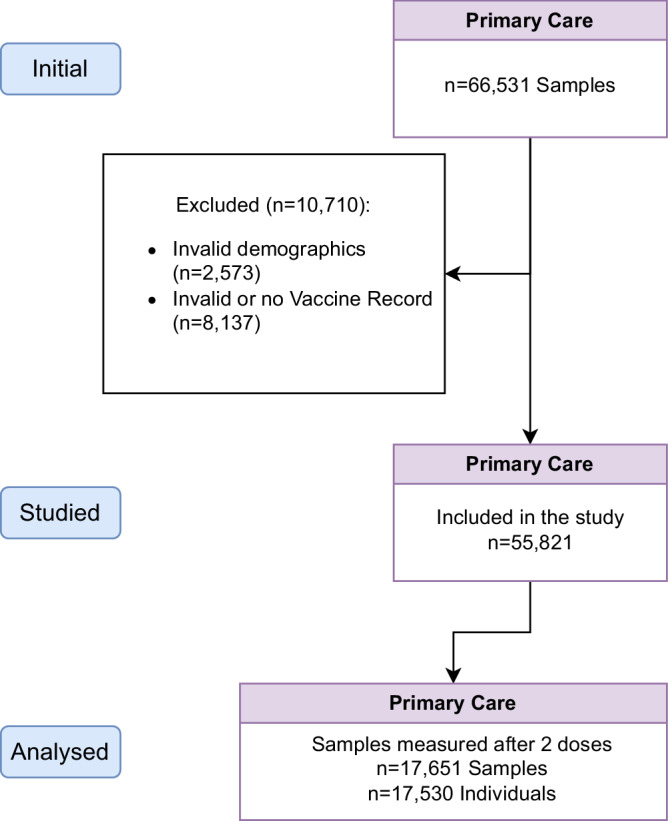
Flow diagram of the number of serology samples included and analysed. After linking these data to EAVE-II, we excluded samples with invalid demographics or lack of vaccine records. Analysis was restricted to samples that were taken at least 14 days after the completion of the primary vaccine course (2 doses).

A total of 66,531 primary care samples were tested for anti-SARS-CoV-2 IgG antibodies between April 20, 2020, and March 28, 2022. (Fig. 1). After excluding samples with missing information (16%, *n* = 10,710) and restricting to those taken at least 14 days after completion of the primary vaccine course (i.e., two doses), 17,651 primary care samples (relating to 17,530 individuals) were available for analysis.

### Descriptive data

Table 1 presents demographic and clinical characteristics overall and for the subset of individuals who tested IgG negative. In total, 7.3% (*n* = 1275) of individuals had at least one negative IgG test recorded after two or more vaccine doses. The percentage who tested IgG negative declined to 0.8% (*n* = 43) (range 0.6–1.0% depending on the vaccine product) after at least three doses of vaccine.

**Table 1:** The number of individuals in the primary care cohort for multiple groupings of covariates – the majority of which were used in at least one of the analyses performed

Covariate	*N* (% of group)	N IgG Negative (% of *N*)
Total	17530 (100)	1275 (7.27)
Age		
0–19	1700 (9.70)	31 (1.82)
20–39	4046 (23.08)	149 (3.68)
40–59	4702 (26.82)	341 (7.25)
60+	7082 (40.40)	754 (10.65)
Sex		
F	8993 (51.30)	627 (6.97)
M	8537 (48.70)	648 (7.59)
BMI		
<18.5	234 (1.33)	16 (6.84)
18.5–25	2573 (14.68)	177 (6.88)
25–30	3584 (20.44)	296 (8.26)
30+	4285 (24.44)	425 (9.92)
Unknown	6854 (39.10)	361 (5.27)
Scottish Index of Multiple Deprivation (SIMD)		
1 – Most Deprived	3331 (19.00)	250 (7.51)
2	3522 (20.09)	273 (7.75)
3	3619 (20.64)	268 (7.41)
4	4013 (22.89)	281 (7.00)
5 – Least Deprived	2938 (16.76)	200 (6.81)
Unknown	3619 (20.64)	268 (7.41)
Immunosuppressed		
No	15186 (86.63)	1004 (6.61)
Yes	1293 (7.38)	104 (8.04)
Severely	1051 (6.00)	167 (15.89)
Advised to shield		
No	15826 (90.28)	1033 (6.53)
Yes	1704 (9.72)	242 (14.2)
Care Home Resident		
No	17401 (99.26)	1259 (7.24)
Yes	129 (0.74)	16 (12.40)
Number of QCOVID Risk Groups		
0	8879 (50.65)	458 (5.16)
1	4733 (27.00)	346 (7.31)
2	2174 (12.40)	206 (9.48)
3-4	1433 (8.17)	201 (14.03)
5+	311 (1.77)	64 (20.58)
QCOVID Risks		
A prior fracture of hip, wrist, spine or humerus	820 (4.68)	87 (10.61)
Atrial Fibrillation	788 (4.50)	103 (13.07)
Asthma	2442 (13.93)	187 (7.66)
Haematological Cancer	144 (0.82)	31 (21.53)
Heart Failure	393 (2.24)	67 (17.05)
Coronary Heart Disease	1349 (7.70)	167 (12.38)
Cirrhosis	173 (0.99)	28 (16.18)
Congenital Heart Disease	258 (1.47)	25 (9.69)
Chronic Kidney Disease	1270 (7.24)	202 (15.91)
Chronic Obstructive Pulmonary Disease	734 (4.19)	87 (11.85)
Cystic Fibrosis or Bronchiectasis or Alveolitis	734 (4.19)	87 (11.85)
Dementia	147 (0.84)	19 (12.93)
Diabetes (Type-I)	168 (0.96)	18 (10.71)
Diabetes (Type-II)	2010 (11.47)	250 (12.44)
Epilepsy	256 (1.46)	18 (7.03)
Rare Neurone Disease	83 (0.47)	17 (20.48)
Pulmonary Hypertension	76 (0.43)	10 (13.16)
Peripheral Vascular Disease	264 (1.51)	36 (13.64)
Rheumatoid Arthritis	523 (2.98)	49 (9.37)
Respiratory Cancer	44 (0.25)	10 (22.73)
Severe Mental Health Illness	2241 (12.78)	197 (8.79)
Sickle Cell Disease	33 (0.19)	6 (18.18)
Stroke	718 (4.10)	100 (13.93)
Thrombosis or Pulmonary Embolus	419 (2.39)	56 (13.37)
Days Since Last Vaccination		
0–49	4285 (24.44)	106 (2.47)
50–99	5440 (31.03)	278 (5.11)
100–149	4307 (24.57)	367 (8.52)
150–199	2487 (14.19)	413 (16.61)
200–300	941 (5.37)	107 (11.37)
>300	70 (0.40)	< 5 ( < 7.04)
Known Prior SARs-CoV-2 Infection		
No	15477 (88.29)	1259 (8.13)
Yes	2053 (11.71)	16 (0.78)
Vaccine Dose		
2 Doses BNT162b2/mRNA-1273	5798 (33.07)	100 (1.72)
2 Doses ChAdOx1	6341 (36.17)	1132 (17.85)
3-4 Mixed Doses (including ChAdOx1)	3109 (17.74)	30 (0.96)
3-4 Mixed Doses (no ChAdOx1)	2282 (13.02)	13 (0.57)
Known Subsequent SARs-CoV-2 Infection after serology test		
No	15252 (87.01)	1055 (6.92)
Yes	2278 (12.99)	220 (9.66)
Subsequent COVID-19 Hospitalisation or Death after serology test		
No	17445 (99.52)	1248 (7.15)
Yes	85 (0.48)	27 (31.76)

The number (and percentage) of those who tested negative for IgG after at least two doses of any COVID-19 vaccines are also shown as a secondary column.

**Note: Rare neurological diseases are motor neurone disease, multiple sclerosis, myaesthenia, or Huntingtons’s chorea.

The percentage of double-vaccinated individuals who tested IgG negative increased with age (3.7% of primary care attendees aged 20–39 tested IgG negative, whereas 10.7% of those aged 60+ tested IgG negative). Among those with no documented clinical risks, 5.2% tested IgG negative, as compared to 20.6% of those with 5 or more risks. Individuals with haematological and respiratory cancers were found to have the highest percentage of individuals who tested IgG negative at 21.5% and 22.7%, respectively.

The most prevalent risk groups were asthma and severe mental health illnesses (Supplementary Fig. 6). Among the primary care cohort attendees with multimorbidity, certain risk groups were more commonly observed with greater multimorbidity and higher mean age. For instance, among those with five or more risks, 66% had coronary heart disease, 59% had type-II diabetes, and 50% had chronic kidney disease (Supplementary Fig. 6). Similarly, among those with five or more risks *and* who had tested IgG negative, the most common risk groups were coronary heart disease (59%), type-II diabetes (59%) and chronic kidney disease (55%) (Supplementary Table 8).

### Characteristics associated with testing IgG negative after completing the primary vaccine course

**Fig. 2:**
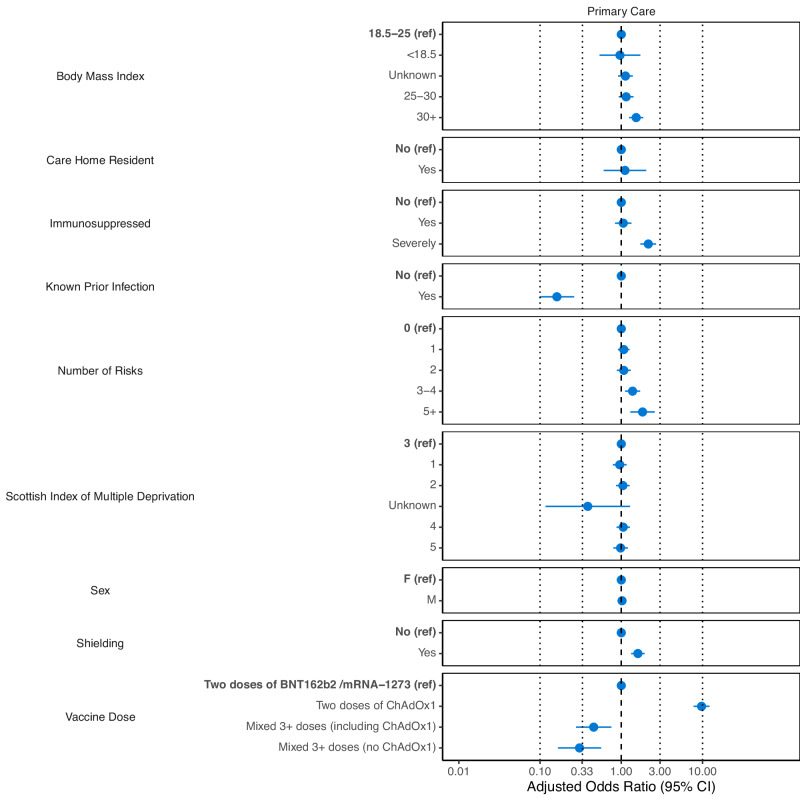
Adjusted odds ratios (ORs) with 95% CIs for testing IgG negative after at least two doses of COVID-19 vaccine for individuals in the primary care cohort (Model A) using the number of risk groups as the primary exposure variable. **Note: (1) additional variables for age, days since vaccination and the calendar period were used and adjusted for in the model (GAM) but are not displayed in this plot. (2) Missing data points indicate that the input variable was not included in the model due to lack of statistical power or was not appropriate.

Individuals with multimorbidity (i.e. those in five or more risk groups) had increased odds (Odds Ratio [OR] 1.94 [95% CI 1.45–2.60]) of testing IgG negative post-vaccination compared to those with no risk group (model A, Table 2, Fig. 2).

**Table 2:** Adjusted and unadjusted odds ratios (ORs with 95% CI) of a negative IgG test result after at least two doses of COVID-19 vaccine for individuals in the primary care cohort (Model A)

Risk Factors (Reference)	Odds Ratios (95% CI)	
	Unadjusted	Adjusted
Care Home Resident (No)		
Yes	1.81 (1.07–3.06)	1.02 (0.55–1.89)
Shielding (No)		
Yes	2.38 (2.05–2.76)	1.68 (1.38–2.04)
Immunosuppressed (No)		
Yes	1.22 (0.99–1.50)	1.05 (0.83–1.33)
Severely	2.66 (2.23–3.17)	2.25 (1.80–2.83)
Prior SARs-CoV-2 Infection (No)		
Yes	0.09 (0.06–0.15)	0.13 (0.08–0.21)
Number of QCOVID Risk Groups (0)		
1	1.44 (1.25–1.67)	1.05 (0.89–1.24)
2	1.93 (1.63–2.29)	1.07 (0.88–1.31)
3-4	2.99 (2.51–3.57)	1.38 (1.11–1.72)
5+	4.72 (3.53–6.31)	1.94 (1.36–2.77)
BMI (18.5–25)		
Unknown	0.75 (0.63–0.91)	1.12 (0.90–1.39)
<18.5	0.99 (0.59–1.69)	0.97 (0.53–1.76)
25-30	1.22 (1.01–1.48)	1.17 (0.94–1.45)
30+	1.49 (1.24–1.79)	1.55 (1.27–1.90)
SIMD (3)		
1	1.02 (0.85–1.22)	0.95 (0.78–1.16)
2	1.06 (0.89–1.26)	1.03 (0.85–1.25)
4	0.95 (0.80–1.13)	1.04 (0.86–1.26)
5	0.92 (0.76–1.11)	1.00 (0.81–1.24)
Unknown	0.36 (0.11–1.15)	0.37 (0.11–1.23)
Sex (Female)		
Male	1.10 (0.98–1.23)	1.06 (0.93–1.20)
Vaccine Dose (2 Doses Pfizer or Moderna)		
Two doses of ChAdOx1	12.38 (10.06–15.24)	9.90 (7.87–12.45)
Mixed 3+ doses (including ChAdOx1)	0.60 (0.40–0.89)	0.68 (0.41–1.12)
Mixed 3+ doses (no ChAdOx1)	0.34 (0.20–0.60)	0.42 (0.23–0.77)

For the adjusted ORs, we additionally adjust for age, days since first measurement and days since last vaccination as splines (see also Figure SB2).

**Fig. 3:**
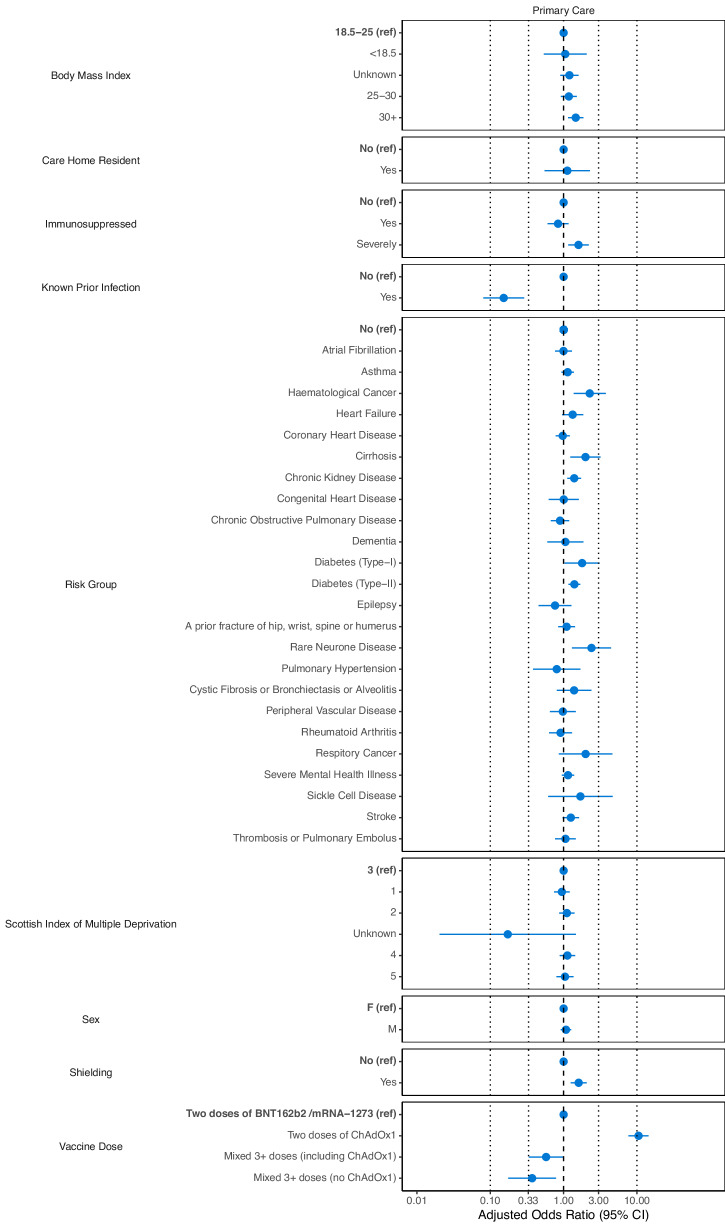
Adjusted odds ratios (ORs) with 95% CIs for testing IgG negative after at least two doses of COVID-19 vaccine for individuals in the primary care cohort (model B) using individual risk groups as the primary exposures. **Note: (1) additional variables for age, days since vaccination and the calendar period were used and adjusted for in the model (GAM) but are not displayed in this plot. (2) Missing data points indicate that the input variable was not included in the model due to lack of statistical power or was not appropriate. (3) ORs for the individual risk groups are calculated compared to individuals who are not in that risk group (as opposed to individuals in no risk groups at all).

For individuals with at least one risk (model B, Table 3, Fig. 3), the following clinical risk factors were associated with increased odds of testing negative: chronic kidney disease (1.38 [1.11–1.72]), cirrhosis (2.19 [1.35–3.57]), Type I diabetes (1.75 [1.00–3.08]), Type II diabetes (1.46 [1.21–1.77]), haematological cancer (2.49 [1.48–4.20]), and rare neurological conditions (2.45 [1.30–4.60]).

**Table 3:** Unadjusted and adjusted odds ratios (ORs with 95% CI) of a negative IgG test result after at least two doses of COVID-19 vaccine for individuals in the primary care cohort with at least one QCOVID risk (model B)

Risk Factors (Reference)	Odds Ratios (95% CI)	
	Unadjusted	Adjusted
Shielding (No)		
Yes	1.88 (1.57–2.25)	1.68 (1.29–2.18)
Immunosuppressed (No)		
Yes	2.19 (1.75–2.74)	1.65 (1.18–2.30)
Severely	0.87 (0.65–1.14)	0.85 (0.61–1.19)
Prior SARs-CoV-2 Infection (No)		
Yes	0.10 (0.05–0.19)	0.13 (0.07–0.24)
QCOVID Risk Group (not in group)		
A prior fracture of hip, wrist, spine or humerus	1.15 (0.91–1.45)	1.04 (0.79–1.37)
Asthma	0.74 (0.63–0.88)	1.12 (0.92–1.38)
Atrial Fibrillation	1.50 (1.20–1.87)	1.07 (0.81–1.40)
Chronic Kidney Disease	2.09 (1.76–2.48)	1.38 (1.11–1.72)
Chronic Obstructive Pulmonary Disease	1.32 (1.04–1.67)	0.93 (0.69–1.25)
Cirrhosis	1.85 (1.23–2.79)	2.19 (1.35–3.57)
Congenital Heart Disease	1.03 (0.68–1.57)	0.93 (0.57–1.52)
Coronary Heart Disease	1.45 (1.21–1.73)	1.00 (0.80–1.26)
Cystic Fibrosis or Bronchiectasis or Alveolitis	1.75 (1.09–2.80)	1.32 (0.76–2.32)
Dementia	1.43 (0.88–2.33)	1.17 (0.66–2.10)
Diabetes (Type-I)	1.14 (0.70–1.88)	1.75 (1.00–3.08)
Diabetes (Type-II)	1.51 (1.29–1.77)	1.46 (1.21–1.77)
Epilepsy	0.72 (0.44–1.16)	0.74 (0.44–1.26)
Haematological Cancer	2.90 (1.97–4.29)	2.49 (1.48–4.20)
Heart Failure	2.03 (1.54–2.67)	1.38 (0.98–1.94)
Peripheral Vascular Disease	1.55 (1.08–2.21)	0.96 (0.63–1.45)
Pulmonary Hypertension	1.47 (0.75–2.86)	0.82 (0.39–1.76)
Rare Neurological Conditions	2.44 (1.43–4.17)	2.45 (1.30–4.60)
Respiratory Cancer	2.86 (1.41–5.81)	1.98 (0.84–4.69)
Rheumatoid Arthritis	0.97 (0.72–1.31)	0.88 (0.61–1.27)
Severe Mental Health Illness	0.90 (0.76–1.06)	1.15 (0.94–1.40)
Sickle Cell Disease	2.00 (0.83–4.84)	1.57 (0.55–4.52)
Stroke	1.63 (1.30–2.04)	1.25 (0.96–1.63)
Thrombosis or Pulmonary Embolus	1.49 (1.12–2.00)	1.08 (0.77–1.51)
BMI (18.5-20)		
Unknown	0.91 (0.70–1.17)	1.23 (0.92–1.65)
<18.5	1.21 (0.66–2.20)	1.12 (0.56–2.25)
25-30	1.21 (0.97–1.52)	1.23 (0.95–1.58)
30+	1.42 (1.15–1.76)	1.48 (1.16–1.90)
SIMD (3)		
1	0.99 (0.79–1.23)	0.95 (0.74–1.22)
2	1.05 (0.84–1.30)	1.08 (0.84–1.38)
4	0.97 (0.77–1.21)	1.13 (0.88–1.45)
5	0.97 (0.76–1.23)	1.06 (0.81–1.40)
Unknown	0.23 (0.03–1.66)	0.19 (0.02–1.50)
Sex (Female)		
Male	1.13 (0.98–1.31)	1.14 (0.96–1.35)
Vaccine Dose (2 doses BNT162b2/mRNA-1273)		
Two doses of ChAdOx1	11.88 (8.85–15.95)	10.99 (7.99–15.13)
Mixed 3+ doses (including ChAdOx1)	0.64 (0.39–1.04)	0.84 (0.49–1.46)
Mixed 3+ doses (no ChAdOx1)	0.39 (0.19–0.81)	0.52 (0.24–1.10)

For the adjusted ORs, we additionally adjust for age, days since first measurement and days since last vaccination as splines (see also Figure SB2).

Increased risks were observed for those who had been advised to remain indoors by the Scottish Government, due to medical fragility, subsequently referred to as “shielding” (1.68 [1.29–2.18]), and those who were severely immunosuppressed (2.25 [1.80–2.83]). Individuals who, at the time of the serology measurements, had received three or more vaccine doses or had at least one known prior SARS-CoV-2 infection, had decreased odds of testing IgG negative (Tables 2 and 3).

In model A, obesity (BMI $\ge$ 30) was associated with an increased risk of testing IgG negative. Neither Scottish Index of Multiple Deprivation (SIMD) nor biological sex were found to have a statistically significant effect on the outcome. We observed the ORs for testing IgG negative increased with age (Supplementary Fig. 2a) and days since last vaccination (Supplementary Fig. 2b). In contrast, we observed a decreasing trend in ORs as a function of time since the first serology measurement (Supplementary Fig. 2c).

We conducted complementary analyses on serology samples extracted from blood donors to provide a comparison population; the results are detailed in Supplementary Note 1, Supplementary Tables 1-4. We conducted sensitivity analyses to examine the effect of including individuals who had received only one dose of any COVID-19 vaccine at the time of the antibody testing (Supplementary Note 2). We found individuals in additional risk groups (sickle cell disease or rheumatoid arthritis) had increased ORs of testing IgG negative after only one dose, but the odds were not significantly greater after completing the primary vaccine course. We also carried out an additional sensitivity analysis where we used more and less conservative definitions of a negative IgG test result; the former being one in which the quantitative IgG measurement could not detect any IgG, and the latter being one deemed to be a measurement of an arbitrary low level of IgG (Supplementary Note 3). The tighter definition yielded significantly higher ORs of a post-vaccination undetectable IgG test result for individuals in increasing number of risk groups (Supplementary Fig. 8), as well as individuals in at least one risk group (Supplementary Fig. 9) with: haematological cancer, pulmonary hypertension, a rare neurological disease, cirrhosis, a history of heart failure, or coronary heath disease.

### Association between IgG levels and subsequent SARS-CoV-2 infection or severe COVID-19 outcomes

Thirteen percent (*n* = 2278) of individuals tested positive for SARS-CoV-2 (at least once) after a serology sample was taken. Risk of SARS-CoV-2 infection (model C, Table 4) was higher amongst post-vaccination IgG negatives, relative to IgG positives (Hazard Ratios [HR] of 1.50 [1.30–1.73]). Administration of additional vaccines between the date of IgG measurement and the outcome reduced the HR (Supplementary Fig. 3). Supplementary Note 1 additionally provides a comparison using samples extracted from blood donors for model C.

**Table 4:** Unadjusted and adjusted hazard ratios (HRs with 95% CIs) of SARS-CoV-2 infection after at least two doses of COVID-19 vaccine for individuals in the primary care cohort (model C)

Variable of Interested (Reference)	Infection Hazard Ratios (95% CI)	
	Unadjusted	Adjusted
**Tested IgG Negative (No)**		
Yes	1.18 (1.03–1.36)	1.50 (1.30–1.73)

ORs were adjusted for: the number of risk groups, age, sex, shielding, care home residency, BMI, SIMD, location and the number of subsequent vaccinations. The positive PCR test rate of double-vaccinated-serology-tested individuals (subsequent infection) was 13.0% of the total primary care attendees (*n* = 2278).

In total, 85 individuals (0.5%) were either hospitalised due to COVID-19 and/or died of a COVID-19-related death. Of those with severe COVID-19 outcomes, 31.8% had previously tested IgG negative after completing the primary vaccine course (Table 1).

The risk of hospitalisation or death was higher for individuals who had tested IgG negative post-vaccination (3.68 [2.28–5.94]) (model D, Table 5). Individuals with five or more risks were 13.37 [6.05–29.53] times more likely to be hospitalised or die from COVID-19 than an individual with no risks.

**Table 5:** Unadjusted and adjusted hazard ratios (HRs with 95% CIs) of hospitalisation or death due to COVID-19 after at least two doses of COVID-19 vaccine for individuals in the primary care cohort (models D and E)

Model	Risk Factors (Reference)	Hospitalisation/Death Hazard Ratios (95% CI)	
		Unadjusted	Adjusted
D	Testing IgG Negative (No)		
	Yes	4.82 (3.07–7.59)	3.68 (2.28–5.94)
	Number of Risk Groups (0)		
	1	1.33 (0.68–2.62)	1.66 (0.82–3.38)
	2	2.86 (1.47–5.56)	3.14 (1.53–6.42)
	3-4	6.23 (3.39–11.44)	5.24 (2.64–10.41)
	5+	16.67 (8.22 – 33.81)	13.37 (6.05 – 29.53)
E	IgG Quantile (High [230-1999 BAU/ml])		
	Undetectable ( < 4.8 BAU/ml)	16.51 (8.74–31.19)	9.21 (4.63 – 18.29)
	Very Low (4.8-33.7 BAU/ml)	2.55 (1.31–4.97)	2.15 (1.08 – 4.26)
	Low (33.8-229 BAU/ml)	1.55 (0.89–2.70)	1.57 (0.89 – 2.77)
	Very High ( ≥ 2000 BAU/ml])	0.42 (0.19–0.93)	0.33 (0.15 – 0.74)
	Haematological Cancer (No)	7.86 (3.43–18.03)	2.79 (1.15 – 6.76)
	Coronary Heart Disease (No)	3.80 (2.36–6.13)	1.64 (0.97 – 2.76)
	Pulmonary Hypertension (No)	13.02 (5.27–32.14)	4.99 (1.95 – 12.75)
	Chronic Kidney Disease (No)	4.90 (3.09–7.77)	2.21 (1.33–3.65)

In model D, the qualitative IgG test result (positive or negative) was used along with the number of risk groups as covariates. In model E, we use quantiles of the antibody IgG levels with high risk (of COVID-19 severe outcomes) groups decoupled from all other risk groups.

ORs were also adjusted for: the number of additional risk groups (model E only), age, sex, BMI and the number of subsequent vaccinations. The hospitalisation/death rate of double-vaccinated-serology-tested individuals (severe outcomes) was 0.5% of the total primary care attendees (*n* = 85).

HRs for model E are also shown in Table 5 (and Supplementary Fig. 4). Compared to those with a high IgG level, the risk of COVID-19 hospitalisation or death was: 9.21 [4.63–18.29] for those with an undetectable IgG level; 2.15 [1.08–4.26] for those with a very low IgG level; 1.57 [0.89–2.77] for those with a low IgG level; and 0.33 [0.15–0.74] for those with a very high IgG level. Of the risk groups, increased risk of COVID-19 hospitalisation or death was observed in individuals with haematological cancer (2.79 [1.15–6.76]), pulmonary hypertension (4.99 [1.95–12.75]), and chronic kidney disease (2.21 [1.33–3.65]).

## Discussion

We found that individuals with multimorbidity were more likely to test IgG negative following at least 2 doses of COVID-19 vaccine. We also found that individuals who tested IgG negative post-vaccination were at greater risk of subsequent SARS-CoV-2 infection (approximately 1.5-fold) and, crucially, COVID-19 hospitalisation or death (nearly 4-fold) compared to those who had tested positive for IgG. In addition, post-vaccination IgG levels were associated with severe COVID-19 outcomes in a dose-dependent manner (i.e., increasing risk with decreasing IgG level), with those having the lowest IgG levels being at the greatest risk (2-fold and 9-fold increased risk among those with very low and undetectable antibodies, respectively, relative to those with high IgG levels).

We investigated the association between post-vaccination antibody response and risk of subsequent severe COVID-19 outcomes at a population level^13,14^. Other studies have examined the association between antibody levels and death from COVID-19, but these have focussed on small cohorts of patients with COVID-19 already admitted to hospital/critical care^15–17^. We demonstrated a dose-response relationship between post-vaccination antibody levels and hospitalisation or death from COVID-19, with decreasing antibody level associated with increasing risk of hospitalisation or death, and highlighting the group of individuals with undetectable post-vaccination IgG as especially vulnerable. We demonstrated that multimorbidity is a risk factor for insufficient IgG responses post-COVID-19 vaccination. We also showed that individuals with obesity, immunosuppression, and those advised to shield during the pandemic were at increased risk of testing IgG negative post-vaccination, as well as individuals with specific clinical risk factors (chronic kidney disease, cirrhosis, Type I diabetes, Type II diabetes, haematological cancer, and rare neurological conditions). Previous studies involving the EAVE-II cohort have identified similar factors (including multimorbidity and underlying health conditions such as individuals receiving immunosuppressants and those with chronic kidney disease) as being higher risk for severe COVID-19 outcomes^13,14^. Our findings suggest that IgG may mediate these associations. Individuals who are more likely to test IgG negative post-vaccination, indicative of a suboptimal post-vaccination immune response, may be eligible for COVID-19 therapeutics or modified vaccination strategies. However, it is notable that modified vaccination strategies, either through extra doses or by considering the timing of doses in relation to treatment, may benefit some, but potentially not all, as there may be persistent non-responders.

As in other studies^12^, our results demonstrated an increase in antibody levels with further vaccine doses, with a very small proportion (0.8%) testing IgG negative after three or more doses. It is possible that the proportion who remain seronegative would decrease even further after 4 or 5 vaccine doses. Among the individuals who had received ≥3 vaccine doses, and still tested IgG negative, 48% (*n* = 23) were in at least two risk groups. Of all individuals who had received ≥2 doses of the vaccine and tested IgG negative, 31.8% (*n* = 27) of these subsequently had severe outcomes.

Given that the data used in our analyses were derived from linkage of national administrative and surveillance datasets, the key strengths of our study include efficiency (using existing data as opposed to collecting primary data, which has significant time and cost implications), large sample sizes, national coverage and reduced risk of bias as they were not subject to the biases that can arise in traditional cohort studies – for example, biases resulting from participant attrition. However, our study also has limitations. Given the small number of individuals in some subgroups (for example individuals testing IgG negative after ≥3 vaccine doses), it was not possible to further stratify the data to provide information on the specific risk groups and vaccines used because of statistical disclosure requirements. There are potential biases in the study population: individuals attending primary care and getting a blood sample taken are more likely to have comorbidities than the general population (48% of individuals in our primary care cohort were in at least one risk group, as compared to 30% in the general population of Scotland). A reasonably large proportion (16%) of potentially eligible records were excluded because of missing information (e.g., on vaccination, risk categories, BMI, etc.). Bias may have been introduced if those with missing information were systematically different regarding the exposure and outcome variables.

Because the serology samples were taken at various time points after vaccination occurred, we do not know whether, among those who tested IgG negative, vaccines never induced seroconversion or whether levels waned over time, although we adjusted our models for time since vaccination to try to account for this. Our study was underpowered to detect differences between those who tested negative shortly after vaccination (i.e. never generated IgG) and those who tested negative at a much later time point (i.e. IgG waned) and future analyses should examine any differences in risk between these two groups. Given we did not follow up individuals over time, we also do not know if subsequent vaccines (e.g., three or more doses) will have induced seroconversion among those who tested negative after two doses. Further, the immune response to vaccination is complex and we have only assessed one component of humoral immunity (anti-spike IgG) and not the other types of humoral (e.g. IgA, IgM, neutralising antibodies) or cellular immunity^18^. For example, T-cells have been shown to play a significant role in protection against severe COVID-19 disease and death^19^, therefore we may be ’misclassifying’ individuals as having suboptimal post-vaccination responses (according to our measure of IgG) when they in fact have adequate T-cell responses. However, previous studies have found that IgG is highly correlated with neutralising antibody activity^4^, which is consistent with our results showing that the presence or levels of IgG are associated with post-vaccination COVID-19 outcomes (i.e., infection, hospitalisation and death), confirming that anti-S IgG is a good overall indicator of immune response. Additionally, S-based assays cannot distinguish between vaccine- or infection-induced antibodies, and we therefore do not know if an individual has additional immunity conferred by prior SARS-CoV-2 infection. We adjusted our models for prior infection; however, COVID-19 infection measured by Reverse Transcription Polymerase Chain Reaction (RT-PCR) may have been an underestimate as it did not include COVID-19 antigen tests done in the community and we will also have missed undiagnosed infections.

Our findings confirm previously reported associations between the following risks or characteristics and a suboptimal antibody response following vaccination (individuals may fall into more than one risk group): cancers^7,20,21^, obesity^7,22,23^, use of immunosuppressant therapies^6,20,21,24,25^, being on the shielded patient list^25^ and age^6,7,26^. Our findings of a greater likelihood of testing negative for antibodies post-vaccination among specific clinical risk groups (those with kidney disease^7,27^, cirrhosis^28^, diabetes^7,29^, haematological cancers^30–32^, and neurological conditions^7,33^) were also consistent with the published literature, although in some of these conditions a diminished immune response may be attributable to therapies used to treat the condition rather than the condition itself. We did not however observe any association between socioeconomic status or sex and antibody response – the latter finding in contrast to previously published results, which have demonstrated higher SARS-CoV-2 IgG levels among females^7,26^. Prior SARS-CoV-2 infection was associated with very low likelihood of testing IgG negative, consistent with previous findings that those who are both infected and vaccinated have greater magnitude and persistence of antibody responses^7,34,35^. We are also aware of several studies that have directly examined the association between antibody responses to vaccination and subsequent SARS-CoV-2 infection^5,8–12^. One of these, a large cohort study (N > 4000) derived from a UK-based twin registry, found that those with the lowest 20% of antibody levels post-vaccination had 3-fold greater odds of SARS-CoV-2 infection^12^.

Our analysis spans a large timeframe, which includes the periods relating to the emergence and dominance of the Delta and Omicron variants. Studies have shown decreased vaccine effectiveness over time as new variants diverge from the original variants that were used to derive the vaccines, which may relate to the specificity of vaccine-induced antibodies^3,36,37^. We did not have information on the SARS-CoV-2 variant for relevant exposures or outcomes; therefore, we adjusted for the time elapsed since beginning of pandemic to account for the prevalence of different variants in Scotland (see Supplementary Fig. 5). However, this approach may not fully account for the risk associated with different variants, and we acknowledge this as a limitation.

Different vaccines have different immunogenic profiles; we attempted to account for this by adjusting for the vaccine product administered. We found that individuals who had received two doses of ChAdOx1 were significantly less likely to test positive for IgG than the reference category (two doses of BNT162b2 or mRNA-1273). This may be partly due to systematic differences among those who received ChAdOx1 because of vaccine programme prioritisation, and other unmeasured confounders; however, it may also be partly attributable to the known lower immunogenicity of the ChAdOx1^38^.

In conclusion, we have demonstrated a strong association between IgG antibody levels and severe COVID-19 outcomes, illustrating the clinical utility of measuring IgG. We have also identified subgroups of the population who generated suboptimal serological responses post-COVID-19 vaccination – particularly those with multimorbidity – and therefore who remain at increased risk of serious COVID-19 outcomes. Consideration should now be given to extend the indications for COVID-19 therapeutics to include those living with multiple long-term conditions, rather than on the basis of single conditions alone. More research into COVID-19 therapeutics for those living with multimorbidity is required.

## Methods

### Study design and population

We used linked data from Early Pandemic Evaluation and Enhanced Surveillance of COVID-19 (EAVE II), a Scotland-wide cohort of 5.4 million people ( ~ 99% of the Scottish population), consisting of primary and secondary care, COVID-19 vaccination, SARS-CoV-2 testing, and mortality data^39^. Additionally, we used SARS-CoV-2 antibody testing data from the Enhanced Surveillance of COVID-19 in Scotland (ESoCiS), a national serological surveillance programme that sampled residual blood from multiple sources, including antenatal, blood donor, paediatric and primary care (i.e. general practice) settings, and tested these for the presence of SARS-CoV-2 IgG antibodies to the spike protein^40,41^.

Serology measurements used in this study were obtained from testing residual blood from people attending primary care settings for routine purposes. Samples were obtained from biochemistry laboratories (700 weekly samples) covering 11 regional health authorities, which represent >90% of the Scottish population. Additionally, primary care samples related to individuals aged 6 years and older and were chosen according to an age/sex/geographical sampling frame proportional to the Scottish general population. The serology results for primary care samples taken between April 20, 2020 and March 28, 2022 were linked with EAVE II data. All analyses were restricted to individuals who had received at least two doses of a COVID-19 vaccine prior to the serology sample date, unless otherwise specified.

We also undertook supplementary analyses of serology measurements from blood donors, similar to those applied to the primary care cohort. Methods and results of these analyses are described in Supplementary Note 1.

This study involves human participants. The Public Benefit and Privacy Panel Committees of Public Health Scotland and Scottish Government approved the linkage and analysis of the deidentified datasets for this project (2021-0115).

### Definition of outcomes

We defined testing IgG negative, to be a negative SARS-CoV-2 IgG test result obtained post-vaccination (at least 14 days after completion of the primary vaccine schedule). Assay manufacturers define a negative IgG test by quantitative IgG levels and stated cut-offs: <33.8 binding antibody units (BAU) per ml (tested using the Diasorin SARS-CoV-2 TrimericS IgG assay for antibodies to the S1/S2 protein)^42^. The outcome of a test result with undetectable IgG levels, as determined by the assay manufacturers, using a cut-off of <4.8 [BAU/ml], is presented in the sensitivity analysis in Supplementary Note 3. An arbitrary cut-off of <100 [BAU/ml] was also used in a sensitivity analysis to study a looser definition of a low post-vaccination IgG response.

For the analysis of the association between SARS-CoV-2 IgG antibody levels (hereafter referred to as “IgG levels”) and subsequent SARS-CoV-2 infection or severe COVID-19 outcomes, we considered SARS-CoV-2 infections, hospitalisations and deaths that occurred after the serology test date. SARS-CoV-2 infections were defined as positive RT-PCR test results; for multiple positive tests, the first test after the serology test was selected. COVID-19 hospitalisations were sourced from the Scottish Morbidity Record and were defined as hospital admissions where COVID-19 was listed as the primary reason for admission (International Classification of Diseases [ICD-10] codes U07.1 and U07.2); COVID-19 deaths were sourced from National Records of Scotland and defined as deaths where COVID-19 was mentioned on the death certificate (also ICD-10 codes U07.1 and U07.2).

### Exposure definitions

For the analysis of factors associated with a negative IgG test, we used 26 comorbidity-based risk groupings^43^. We used either the total number of risk groups (0, 1, 2, 3-4, ≥5) for all individuals, or the specific risk groups as separate variables (Supplementary Table 5).

For the analysis of IgG levels and subsequent COVID-19 outcomes, the exposure was defined as either a positive or negative IgG test result, or as quantiles of the IgG. Quantiles were defined by analysis of the distribution of all measurements (Supplementary Fig. 1) as: very high (≥2000 BAU/ml), high (230–1999 BAU/ml), low (33.8–229 BAU/ml), very low (4.8–33.7 BAU/ml), and undetectable (< 4.8 BAU/ml).

### Confounding factors

In all analyses, we accounted for potential confounding due to: biological sex; age; BMI; SARS-CoV-2 infection (i.e. prior to the serology sample date); vaccine dose and vaccine product last administered ( ≥ 14 days prior to the serology sample date); number of days between the last vaccine dose and the serology sample date; number of days since the start of the pandemic (March 1, 2020); classification variables – derived from vaccine records and patient data held by the national public health body relating to whether the individual was a care home resident, immunosuppressed, severely immunosuppressed, and/or on the Scottish shielded patient list; and socio-economic status measured in quintiles of SIMD.

In the analysis investigating the association between IgG levels and COVID-19 related outcomes, either the total number of risk groups (0, 1, 2, 3-4, ≥5), or specific risks associated with severe outcomes were included as confounders. We accounted for the administration of additional vaccine doses (or boosters) between the serology sample date and the COVID-19 related outcome. We also adjusted for Urban Rural Classification (URC) and SIMD.

### Statistical analysis

**Fig. 4:**
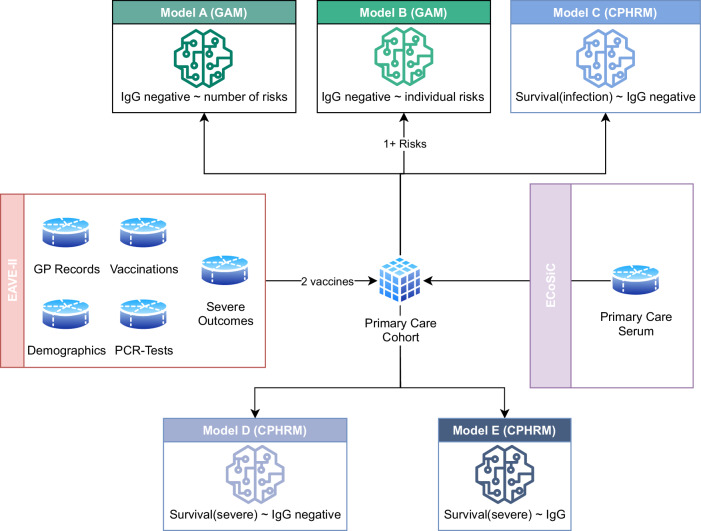
Illustrative diagram of all models that were constructed and used for analysis in this paper. Given input datasets from EAVE-II and serology datasets from ESoCiS we constructed data containing serology measurements linked with individual demographics, GP records, SARs-COV-2 testing and hospitalisation data for individuals who had received at least two doses of any COVID-19 vaccine. Model A and Model B are Generalised Additive Models (GAMs) where the number of comorbidity-based risks is used as the exposure for the outcome of testing IgG negative after vaccination. Similarly Model B instead used individual risks as the exposure variable for all individuals with at least one risk. Models C, D and E are Cox Proportional Hazard Regression Models (CPHRMs) where the survival is defined as SARs-CoV-2 infection for Model C and hospitalisation/death for Models D and E. Testing negative for IgG is the exposure for models C, and D whereas quantiles of quantitative IgG measurements are used as the exposure for model E.

To examine characteristics associated with testing IgG negative post-double vaccination, we fitted two generalised additive models (GAMs) (Fig. 4). To examine multimorbidity, model A included the total number of risk groups (e.g., 1, 2, 3-4, or 5 + ) as an exposure variable; model B included specific risk groups (e.g., asthma, chronic kidney disease, etc.) as exposure variables and were restricted to individuals with at least one risk. For more details of each specific model, please see Supplementary Table 7.

GAMs were used to account for the influence of multiple confounding variables, some of which exhibited non-linear relationships. Smoothed spline functions were used to address such confounding due to age, the time interval between the last vaccine administration and the serology sample date, as well as the date of the serology sample, to account for residual effects due to the period of the pandemic. Age was stratified by number of vaccine doses, type of product and product combinations, motivated by the timeline and prioritisation of vaccine rollout in Scotland.

Additional risk factors were chosen for inclusion in the models if the p-value calculated in a univariate model was 0.1, or if there was a clinical rationale for their inclusion (and if the group had five or more individuals with the outcome). We calculated adjusted (and unadjusted) ORs from the exponential of the coefficients of the parametric terms of the GAM, using a Wald statistic for the confidence intervals (at 95% CI).

To study the association between IgG levels and subsequent SARS-CoV-2 infection or severe COVID-19 outcomes, we fitted a further two models: one using infection as the outcome (model C), and two with severe COVID-19 as the outcome (models D and E). In model D, the number of risk groups was included as a confounder while in model E, individual risks that were associated with significantly higher risk of severe outcomes were decoupled from all other risk groups and included separately as confounders. In models C and D, we used a binary exposure variable (positive/negative IgG test result) with the number of risk groups as a confounder. In Model E, we used quantiles of IgG titres as the exposure to further investigate how IgG levels, rather than just positive/negative IgG tests, affected the HRs of severe outcomes. These models are also summarised in Supplementary Table 7.

The number of vaccinations post-serology sample date were adjusted for as a time-dependent covariate. Age, sex, and BMI were adjusted for in all models (C, D, and E), with the survival period defined with respect to the start of the pandemic, accounting for the severity of SARS-CoV-2 variants, virus abundance in the general population, lockdowns, and other changes in social relations. Further adjustments were made for care home residency, SIMD, and urban/rural classification in Model C.

The follow-up period extended up to three months after the last recorded IgG measurement (i.e., up to June 28, 2022). Individuals were censored if they died from non-COVID-19 reasons before the end of the study period. Hazard ratios (HR) were calculated from the exponent of the fitted coefficients of the Cox models, with 95% CIs obtained using Wald tests.

All statistical analyses were performed using R version 3.6.1. We followed a pre-specified study analysis plan (available from the authors on request). Results are reported according to the Strengthening the Reporting of Observational Studies in Epidemiology (STROBE) guidelines (Supplementary Table 6).

### Reporting summary

Further information on research design is available in the Nature Research Reporting Summary linked to this article.

### Supplementary information

Supplementary Material

Reporting summary

## Data Availability

The data cannot be shared publicly because it contains potentially identifiable and sensitive patient information and is legally restricted by Public Health Scotland and the Scottish and UK governments. Data were available for researchers who meet the criteria for access due to undertaking this work as part of a national Scottish surveillance program.

## Code availability

A data dictionary covering the datasets used in this study can be found at https://eave-ii.github.io/data-dictionary/. All code used in this study is publicly available at https://github.com/EAVE-II/Serology-Analysis/tree/master/analysis. The data used to undertake these analyses are not publicly available because they are based on deidentified national clinical records. These data are available, subject to approval by the NHS Scotland Public Benefit and Privacy Panel, by application through the Scotland National Safe Haven.
