# Supplementary material for: Association between antibody responses post-vaccination and severe COVID-19 outcomes in Scotland
Source: NPJ Vaccines. 2024 Jun 14;9:107. doi: 10.1038/s41541-024-00898-w (PMC11178861; doi:10.1038/s41541-024-00898-w)
Supplement: Supplementary file 1 — Supplementary Material [file 41541_2024_898_MOESM1_ESM.pdf]

# Supplementary Material

## Supplementary Note 1

We conducted similar analyses using serology measurements obtained from testing residual blood from blood donors. Blood samples obtained from blood donor clinics (500 weekly samples) covering 12 regional health authorities in Scotland were used. Individuals who had donated blood were aged 17 years or older and samples were taken between April 20, 2020 and March 28, 2022. As with the primary care samples, these were linked with EAVE II data and restricted to individuals who had received at least two doses of a COVID-19 vaccine prior to the serology sample date.

A total of 46,227 blood donor samples were tested for anti-SARS-CoV-2 IgG antibodies between April 20, 2020, and March 28, 2022. After excluding samples with missing information (6%, n=2,930) and restricting to those taken at least 14 days after completion of the primary vaccine course (i.e., two doses), 18,499 blood donor samples (relating to 16,865 individuals) were available for analysis. Blood donor serology samples were measured by Enzyme-linked immunosorbent assay (ELISA). A cut-off of S/o of IgG <1.1 defined a negative IgG test.

We present results obtained for models A, B, and C when using the blood donor cohort instead. Results were not obtainable for models D and E due to a lack of statistical power.

**Supplementary Table 1**

The number of individuals in the blood donor cohort for multiple groupings of covariates – the majority of which were used in at least one of the analyses performed. The number (and percentage) of those who tested negative for IgG after at least two doses of any COVID-19 vaccines are also shown as a secondary column.

**Note:

(1) All numbers that are <5 are removed due to data disclosure laws. When there is no or missing data, entries are marked with a dash.

(2) Rare neurological diseases are Motor neurone disease, multiple sclerosis, myaesthenia, or Huntingtons's Chorea

| **Covariate** | **Blood Donors N (% of group)** | **N IgG Negative (% of N)** |
|---|---|---|
| **Total** | 16865 (100) | 139 (0.82) |
| **Age** | | |
| 0-19 | 654 (3.88) | <5 (<0.76) |
| 20-39 | 5103 (30.26) | 18 (0.35) |
| 40-59 | 8045 (47.70) | 71 (0.88) |
| 60+ | 3063 (18.16) | 46 (1.50) |
| **Sex** | | |
| F | 9235 (54.76) | 64 (0.69) |
| M | 7630 (45.24) | 75 (0.98) |
| **BMI** | | |
| <18.5 | 68 (0.40) | - |
| 18.5-25 | 2519 (14.94) | 17 (0.67) |
| 25-30 | 3575 (21.20) | 31 (0.87) |
| 30+ | 2913 (17.27) | 43 (1.48) |
| Unknown | 7790 (46.19) | 48 (0.62) |
| **Scottish Index of Multiple Deprivation (SIMD)** | | |
| 1 – Most Deprived | 1716 (10.17) | 16 (0.93) |
| 2 | 2607 (15.46) | 32 (1.23) |
| 3 | 3275 (19.42) | 20 (0.61) |
| 4 | 4233 (25.10) | 29 (0.69) |
| 5 – Least Deprived | 4951 (29.36) | 42 (0.85) |
| Unknown | 3275 (19.42) | 20 (0.61) |
| **Immunosuppressed** | | |
| No | 16798 (99.60) | 138 (0.82) |
| Yes | 50 (0.30) | <5 (<9.26) |
| Severely | 17 (0.10) | - |
| **Advised to shield** | | |
| No | 16805 (99.64) | 137 (0.82) |
| Yes | 60 (0.36) | <5 (<7.94) |
| **Care Home Resident** | | |
| No | 16865 (100.00) | 139 (0.82) |
| Yes | - | - |
| **Number of QCOVID Risk Groups** | | |
| 0 | 12770 (75.72) | 86 (0.67) |
| 1 | 3533 (20.95) | 41 (1.16) |
| 2 | 509 (3.02) | 9 (1.77) |
| 3-4 | 53 (0.31) | <5 (<9.09) |
| 5+ | - | - |
| **QCOVID Risks** | | |
| A prior fracture of hip, wrist, spine or humerus | 547 (3.24) | 7 (1.28) |
| Atrial Fibrillation | 23 (0.14) | - |
| Asthma | 1944 (11.53) | 16 (0.82) |
| Haematological Cancer | <5 (<0.03) | <5 (<50) |
| Heart Failure | <5 (<0.03) | - |
| Coronary Heart Disease | 13 (0.08) | - |
| Cirrhosis | 144 (0.85) | <5 (<3.42) |
| Congenital Heart Disease | 38 (0.23) | - |
| Chronic Kidney Disease | 58 (0.34) | <5 (<8.2) |
| Chronic Obstructive Pulmonary Disease | 50 (0.30) | <5 (<9.62) |
| Cystic Fibrosis or Bronchiectasis or Alveolitis | 50 (0.30) | <5 (<9.62) |
| Dementia | - | - |
| Diabetes (Type-I) | <5 (<0.03) | - |
| Diabetes (Type-II) | 240 (1.42) | 11 (4.58) |
| Epilepsy | 65 (0.39) | <5 (<7.25) |
| Rare Neurone Disease | <5 (<0.03) | - |
| Pulmonary Hypertension | - | - |
| Peripheral Vascular Disease | 13 (0.08) | - |
| Rheumatoid Arthritis | 30 (0.18) | <5 (<14.71) |
| Respiratory Cancer | <5 (<0.03) | - |
| Severe Mental Health Illness | 1401 (8.31) | 21 (1.5) |
| Sickle Cell Disease | <5 (<0.03) | - |
| Stroke | 8 (0.05) | - |
| Thrombosis or Pulmonary Embolus | 92 (0.55) | <5 (<5.21) |
| **Days Since Last Vaccination** | | |
| 0 - 49 | 4484 (26.59) | <5 (<0.11) |
| 50 - 99 | 5831 (34.57) | 24 (0.41) |

| | | |
|---|---|---|
| 100 - 149 | 3976 (23.58) | 53 (1.33) |
| 150 - 199 | 2069 (12.27) | 51 (2.46) |
| 200 - 300 | 486 (2.88) | 6 (1.23) |
| >300 | 19 (0.11) | <5 (<21.74) |
| **Known Prior SARs-CoV-2 Infection** | | |
| No | 14844 (88.02) | 135 (0.91) |
| Yes | 2021 (11.98) | <5 (<0.25) |
| **Vaccine Dose** | | |
| 2 Doses BNT162b2/mRNA-1273 | 5606 (33.24) | 21 (0.37) |
| 2 Doses ChAdOx1 | 5771 (34.22) | 115 (1.99) |
| 3-4 Mixed Doses (including ChAdOx1) | 2819 (16.72) | <5 (<0.18) |
| 3-4 Mixed Doses (no ChAdOx1) | 2669 (15.83) | - |
| **Known Subsequent SARs-CoV-2 Infection after serology test** | | |
| No | 14036 (83.22) | 97 (0.69) |
| Yes | 2829 (16.77) | 42 (1.48) |
| **Subsequent COVID-19 Hospitalisation or Death after serology test** | | |
| No | 16856 (99.95) | - |
| Yes | 9 (0.05) | - |

**Supplementary Table 2**

Adjusted and unadjusted odds Ratios (ORs with 95% CI) of a negative IgG test result after at least two doses of COVID-19 vaccine for individuals in the blood donor cohorts (Model A). For the adjusted ORs, we additionally adjust for age, days since first measurement and days since last vaccination as splines (see also Supplementary Figure 2). Due to lack of statistical power some variables were not included in Model A for the blood donors and therefore are shown as blank in the table.

| **Risk Factors (Reference)** | **Blood Donors**<br>**Odds Ratios (95% CI)** | |
|---|---|---|
| | **Unadjusted** | **Adjusted** |
| **Care Home Resident (No)** | | |
| Yes | - | - |
| **Shielding (No)** | | |
| Yes | 4.18 (1.01 - 17.24) | 1.66 (0.32 - 8.45) |
| **Immunosuppressed (No)** | | |
| Yes | - | - |
| Severely | - | - |
| **Prior SARs-CoV-2 Infection (No)** | | |
| Yes | 0.21 (0.08 - 0.57) | 0.27 (0.10 - 0.74) |
| **Number of QCOVID Risk Groups (0)** | | |
| 1 | 1.73 (1.19 - 2.52) | 1.56 (1.04 - 2.32) |
| 2 | 2.67 (1.34 - 5.34) | 2.16 (1.04 - 4.52) |
| 3-4 | 8.87 (2.72 - 28.91) | 9.23 (2.37 - 35.87) |
| 5+ | - | - |
| **BMI (18.5-25)** | | |
| Unknown | 0.91 (0.52 - 1.58) | 0.94 (0.53 - 1.67) |
| <18.5 | - | - |
| 25-30 | 1.27 (0.70 - 2.30) | 1.02 (0.55 - 1.87) |
| 30+ | 2.17 (1.23 - 3.81) | 1.77 (0.98 - 3.16) |
| **SIMD (3)** | | |
| 1 | 1.55 (0.80 - 3.00) | 1.61 (0.82 - 3.18) |
| 2 | 2.02 (1.15 - 3.54) | 2.20 (1.24 - 3.90) |
| 4 | 1.11 (0.63 - 1.97) | 1.16 (0.65 - 2.07) |
| 5 | 1.39 (0.81 - 2.37) | 1.40 (0.81 - 2.41) |
| Unknown | - | - |
| **Sex (Female)** | | |
| Male | 1.41 (1.01 - 1.97) | 1.15 (0.81 - 1.63) |
| **Vaccine Dose (2 Doses Pfizer or Moderna)** | | |
| Two doses of ChAdOx1 | 5.41 (3.39 - 8.62) | 5.13 (3.01 - 8.73) |
| Mixed 3+ doses (including ChAdOx1) | 0.22 (0.07 - 0.75) | 0.35 (0.06 - 2.19 |
| Mixed 3+ doses (no ChAdOx1) | - | - |

**Supplementary Table 3**

Unadjusted and adjusted odds Ratios (ORs with 95% CI) of a negative IgG test result after at least two doses of COVID-19 vaccine for individuals in the blood donor cohorts with at least one QCOVID risk (model B). For the adjusted ORs, we additionally adjust for age, days since first measurement and days since last vaccination as splines (see Supplementary Figure 2). Due to lack of statistical power some variables were not included in the blood donors model B and therefore are shown as blank in the table.

| Risk Factors (Reference) | Blood Donors Odds Ratios (95% CI) | |
|---|---|---|
| | **Unadjusted** | **Adjusted** |
| **Immunosuppressed (No)** | | |
| Yes | 4.17 (0.55 - 31.66) | 1.59 (0.16 - 15.45) |
| Severely | - | - |
| **Prior SARs-CoV-2 Infection (No)** | | |
| Yes | 0.14 (0.02 - 1.02) | 0.15 (0.02 - 1.13) |
| **QCOVID Risk Group (not in group)** | | |
| A prior fracture of hip, wrist, spine or humerus | 0.99 (0.45 - 2.21) | 1.29 (0.49 - 3.41) |
| Asthma | 0.48 (0.27 - 0.87) | 0.90 (0.42 - 1.94) |
| Atrial Fibrillation | - | - |
| Chronic Kidney Disease | - | - |
| Chronic Obstructive Pulmonary Disease | - | - |
| Cirrhosis | - | - |
| Congenital Heart Disease | - | - |
| Coronary Heart Disease | - | - |
| Cystic Fibrosis or Bronchiectasis or Alveolitis | - | - |
| Dementia | - | - |
| Diabetes (Type-I) | - | - |
| Diabetes (Type-II) | 4.19 (2.13 - 8.23) | 3.97 (1.60 - 9.83) |
| Epilepsy | - | - |
| Haematological Cancer | - | - |
| Heart Failure | - | - |
| Peripheral Vascular Disease | - | - |
| Pulmonary Hypertension | - | - |
| Rare Neurological Conditions | - | - |
| Respiratory Cancer | - | - |
| Rheumatoid Arthritis | - | - |
| Severe Mental Health Illness | 1.26 (0.72 - 2.19) | 1.39 (0.68 - 2.83) |
| Sickle Cell Disease | - | - |
| Stroke | - | - |
| Thrombosis or Pulmonary Embolus | | |
| **BMI (18.5-20)** | | |
| Unknown | 0.23 (0.06 - 0.84) | 0.21 (0.05 - 0.81) |
| <18.5 | - | - |
| 25-30 | 1.14 (0.52 - 2.49) | 0.84 (0.36 - 1.95) |
| 30+ | 1.43 (0.67 - 3.03) | 0.94 (0.41 - 2.18) |
| **SIMD (3)** | | |
| 1 | 1.61 (0.56 - 4.63) | 1.51 (0.50 - 4.60) |
| 2 | 2.13 (0.85 - 5.37) | 2.31 (0.88 - 6.07) |
| 4 | 0.75 (0.26 - 2.14) | 0.73 (0.25 - 2.17) |
| 5 | 1.92 (0.80 - 4.60) | 1.86 (0.75 - 4.64) |
| Unknown | - | - |
| **Sex (Female)** | | |
| Male | 1.25 (0.72 - 2.14) | 0.92 (0.50 - 1.71) |
| **Vaccine Dose (2 doses BNT162b2/mRNA-1273)** | | |
| Two doses of ChAdOx1 | 7.44 (3.17 - 17.49) | 5.77 (2.29 - 14.50) |
| Mixed 3+ doses (including ChAdOx1) | 0.26 (0.03 - 2.18) | 0.46 (0.03 - 7.34) |
| Mixed 3+ doses (no ChAdOx1) | - | - |

**Supplementary Table 4**

Unadjusted and adjusted Hazard Ratios (HRs with 95% CIs) of SARS-CoV-2 infection after at least two doses of COVID-19 vaccine for individuals in blood donor cohorts (model C). ORs were adjusted for: the number of risk groups, age, sex, shielding, BMI, SIMD, location and the number of subsequent

vaccinations. The positive PCR test rate of double-vaccinated-serology-tested individuals (subsequent infection) was 1.5% for blood donors (n=2829).

| | **Infection Hazard Ratios (95% CI) Blood Donors Cohort** | |
|---|---|---|
| Variable of Interested (Reference) | **Unadjusted** | **Adjusted** |
| **Tested IgG Negative (No)** | | |
| Yes | 1.72 (1.26 - 2.33) | 1.93 (1.42 - 2.64) |

## Supplementary Figure 1

a) Distributions of the measured quantitative IgG levels showing positive (green) and negative (red) test results determined by the assays. The top row shows measurements taken on the primary care serology samples; the bottom row shows measurements taken on the blood donor serology samples. Note that different assays were used for the two cohorts, therefore the IgG scale is measured in different units (BAU/ml for the primary care, and arbitrary uncalibrated units for the blood donors). The three columns split measurements by vaccine dose (2, 3, 4+) at the time of serum extraction.

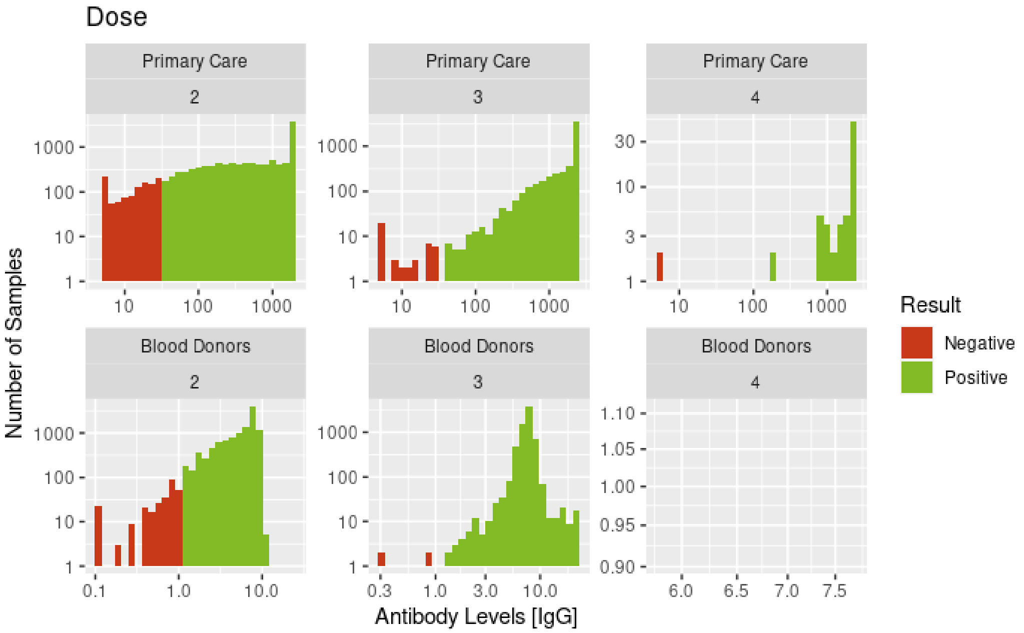


b) The number of available serology samples with certain bins of quantitative IgG measurements (BAU/ml) collected for the primary care cohort. The distribution is coloured by the quantiles used in modelE as the predictor variable: light blue shows IgG levels that were undetectable (<4.8 BAU/ml), grey shows levels that were detected but were not sufficient for a positive test results (>4.8, <33.8 BAU/ml), purple are positive IgG test results with low levels of IgG (>33.8, <230 BAU/ml), dark blue are positive IgG test results with high levels of IgG (>230,<200 BAU/ml) and green are positive tests where the IgG level maxed out the measurement assay at very high levels of IgG and are therefore truncated (>2000 BAU/ml).

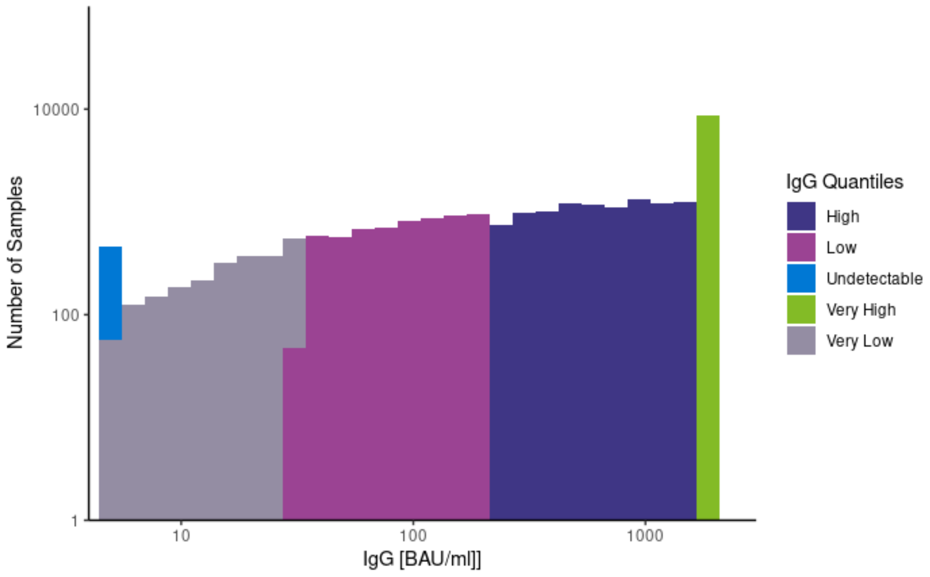
10000
100
1
Number of Samples
10
100
1000
IgG [BAU/ml]]
IgG Quantiles
High
Low
Undetectable
Very High
Very Low

## Supplementary Figure 2

Adjusted odds ratios (ORs) with 95% confidence intervals, for all individuals in both the primary care and blood donor cohorts. ORs were calculated from fitted spline functions, given a reference (indicated by a dashed line on the y-axis, for the variable of interest). S1a) shows the ORs calculated by age, for the primary care cohort, stratified by which vaccine dose was last administered (at the time of the serology measurement). Similarly, but for both cohorts, S1b) shows ORs calculated from the spline for the number of days between the start of the pandemic and the date of the serology measurement (pandemic period), that attempts to account for seasonal and pandemic effects. S3c) shows ORs for the number of days since the last vaccine dose, to account for waning effects.

### a) Age (primary care cohort)

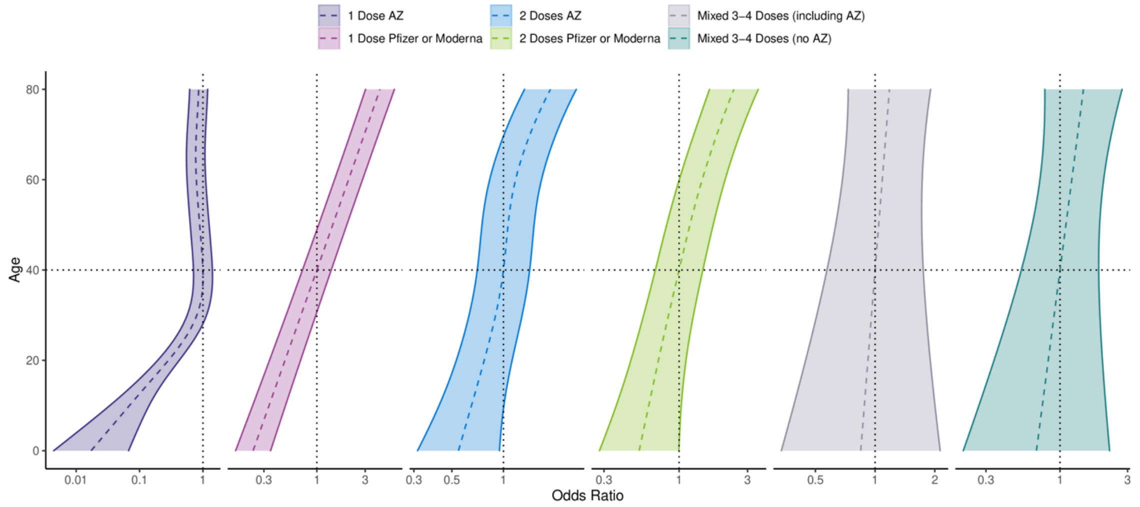


### b) Days since last vaccination

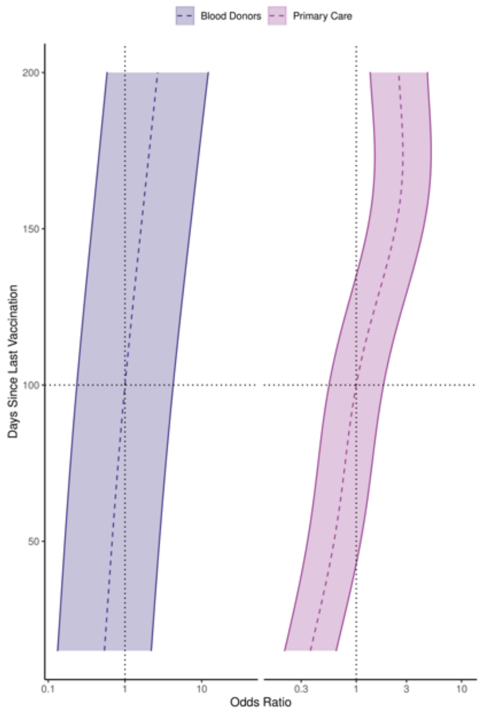


### c) Pandemic Period

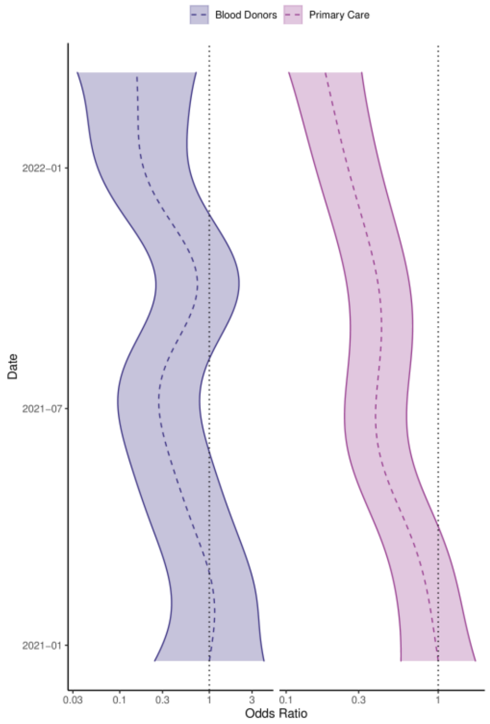

**Supplementary Figure 3**

Adjusted hazard ratios (HRs) with 95% CIs of testing positive for SARs-CoV-2 for all individuals in the Primary Care Cohort (red, left) and the Blood Donors Cohort (blue, right) who were infected at least once after a serology measurement was taken.

**Note:

(1) Missing data points indicate that the input variable was not included in the model due to lack of statistical power or was not appropriate.

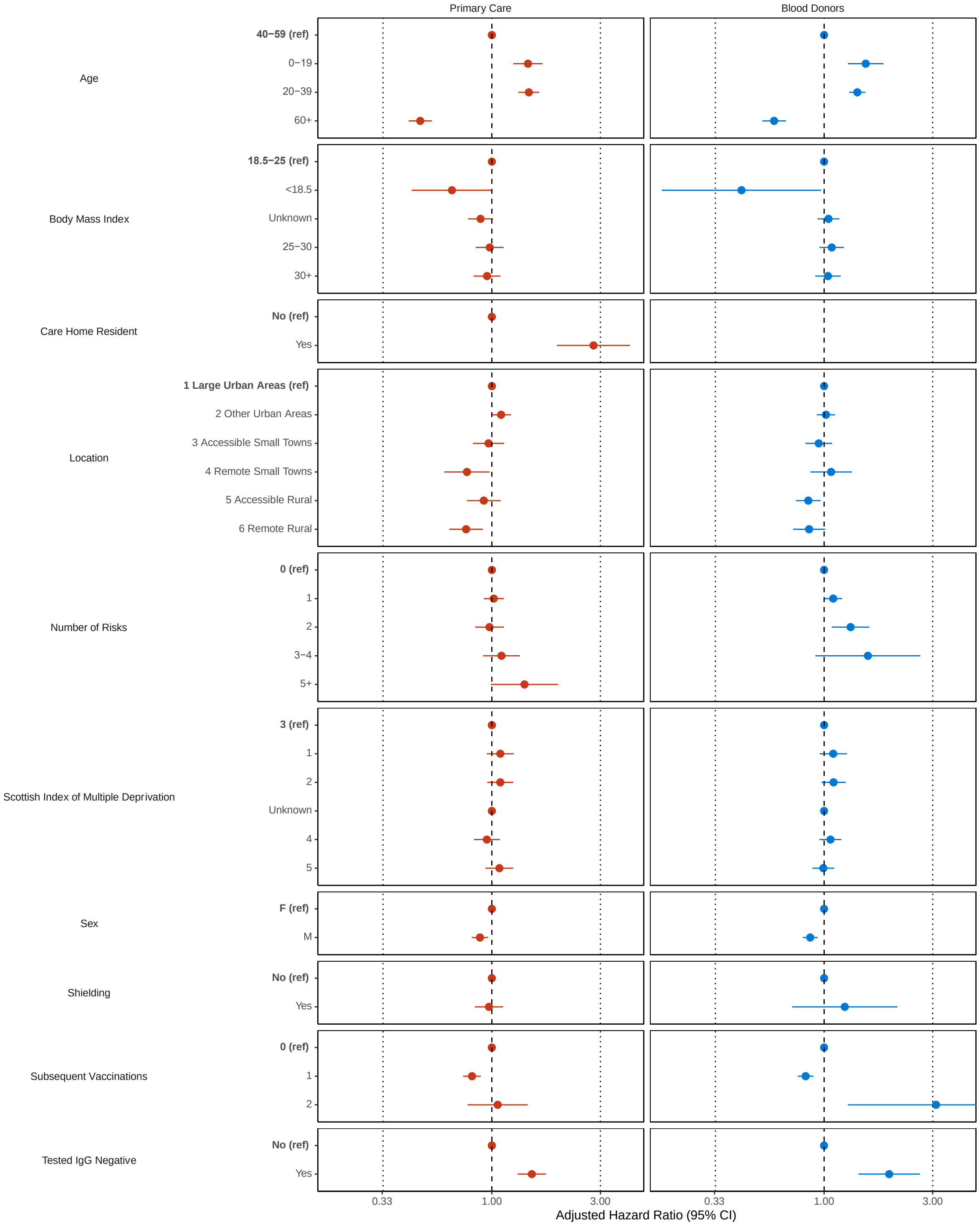

## Supplementary Figure 4

Adjusted hazard ratios (HRs) with 95% CIs all individuals in the primary care cohort who were hospitalised or died due to COVID-19 after a serology measurement was taken. Using the number of risk groups and a binary exposure variable for the antibody measurement (insufficient/sufficient) is shown for model D (left, blue). The HRs using quantiles of the antibody measurements and specific high-risk groups decoupled from all other groups shown on the right (red) for model E.

**Note:
(1) Missing data points indicate that the input variable was not included in the model (as other variables were used).

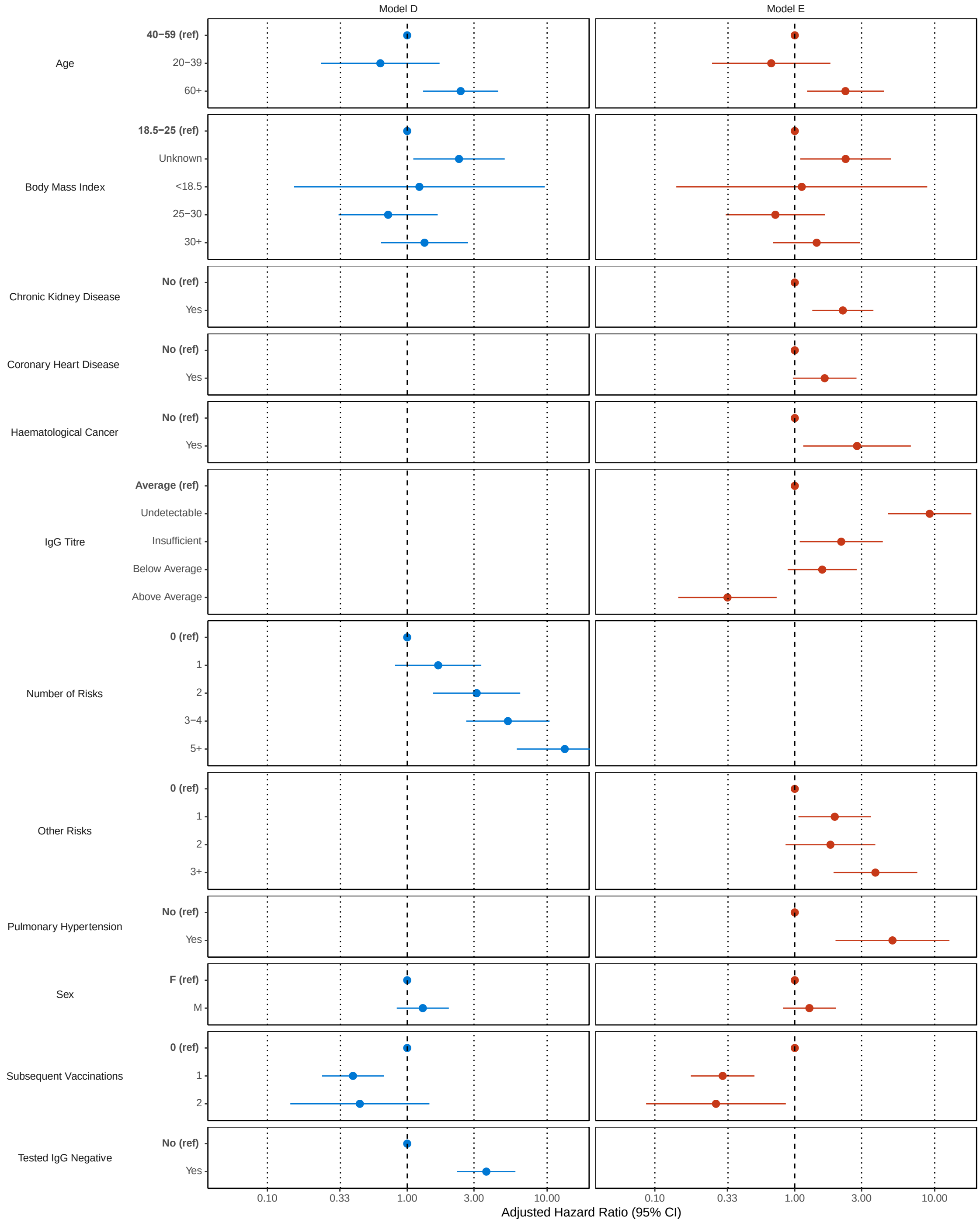

## Supplementary Figure 5

Baseline Hazard Function Plot for SARs-CoV-2 Infection Risk Over Time. The estimated hazard rate of SARs-CoV-2 infection over the duration of the pandemic, with time measured since the start of the outbreak. It provides insights into the temporal patterns of infection risk and the underlying dynamics influencing its occurrence that were accounted for in model C using primary care attendees (blue) and model $C_{BD}$ using blood donors (red). By considering the reference levels of all covariates, the plot contributes to understanding the baseline risk of SARs-CoV-2 infection and its potential associations with various factors.

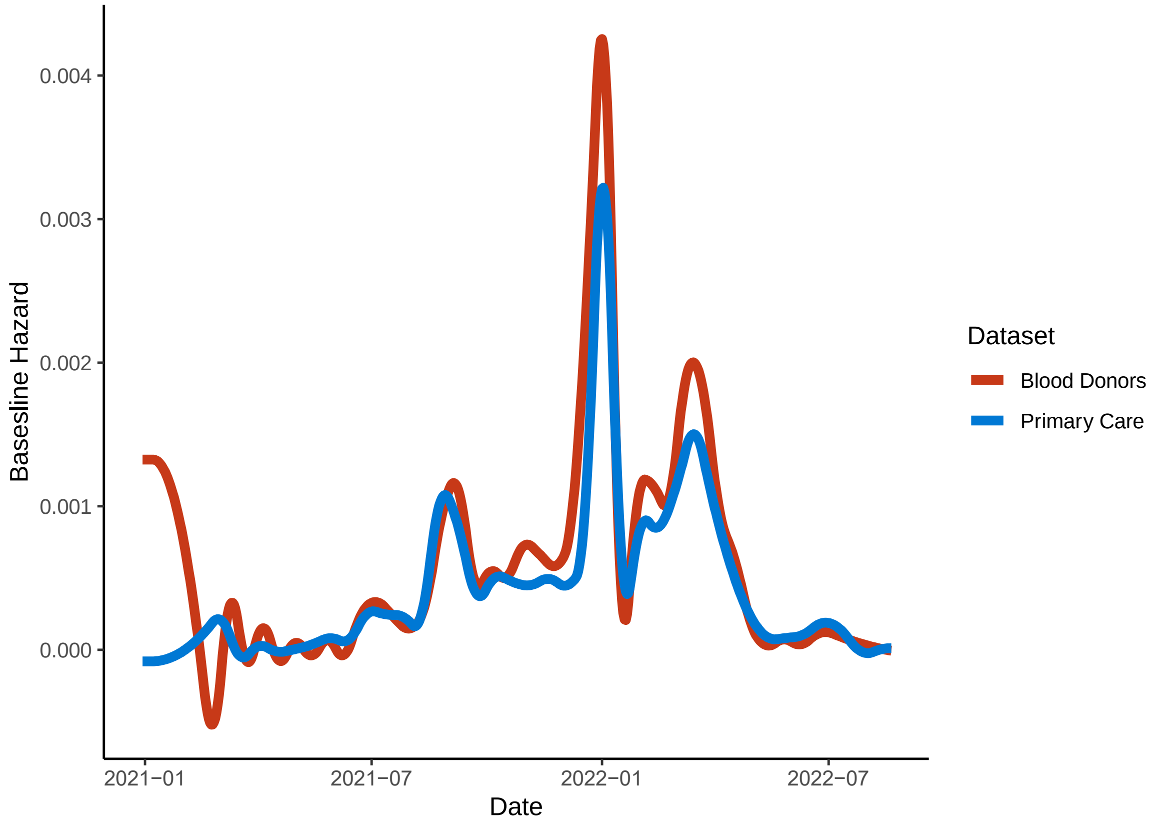

## Supplementary Figure 6

The number of individuals in the primary care cohort who were classified in certain clinical risk groups, stratified both by age group and by the total number of risk groups they were in.

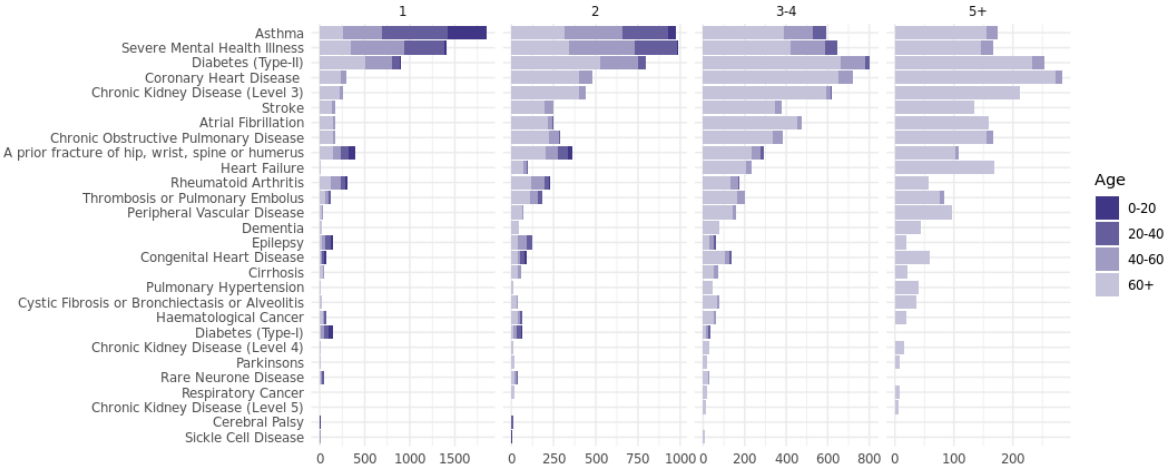


## Supplementary Figure 7

The number of individuals in the blood donor cohort who were classified in certain clinical risk groups, stratified both by age group and by the total number of risk groups they were in.

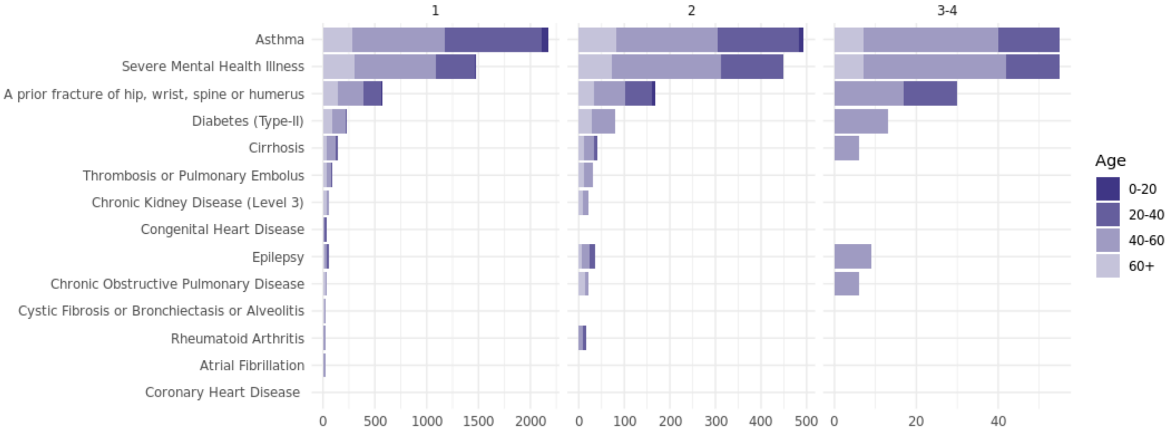

**Supplementary Table 5**

| QCOVID Risk Groups |
|---|
| Atrial fibrillation |
| Asthma |
| Cerebral palsy |
| Coronary heart disease |
| Cirrhosis |
| Congenital heart disease |
| Chronic kidney disease |
| Chronic obstructive pulmonary disease |
| Dementia |
| Diabetes Type 1 |
| Diabetes Type 2 |
| Epilepsy |
| Fracture |
| Haematological cancer |
| Heart failure |
| Neurological disorder |
| Parkinson’s |
| Pulmonary hypertension |
| Pulmonary rare |
| Peripheral vascular disease |
| Rheumatoid arthritis or SLE |
| Respiratory cancer |
| Severe mental illness |
| Sickle cell disease |
| Stroke |
| Thrombosis or pulmonary embolus |

More information on codes: https://github.com/EAVE-II/EAVE-II-data-dictionary
Ref: Clift, A.K., et al. Living risk prediction algorithm (QCOVID) for risk of hospital admission and mortality from coronavirus 19 in adults: national derivation and validation cohort study. BMJ 371,m3731 (2020).

**Supplementary Table 6**

| | Item No | Recommendation | Comments | Location in the manuscript |
|---|---|---|---|---|
| **Title and abstract** | 1 | (*a*) Indicate the study's design with a commonly used term in the title or the abstract | | P1-2 |
| | | (*b*) Provide in the abstract an informative and balanced summary of what was done and what was found | | P1-2 |
| **Introduction** | | | | |
| Background/rationale | 2 | Explain the scientific background and rationale for the investigation being reported | Also in the referenced statistical analysis plan | P4 |
| Objectives | 3 | State specific objectives, including any prespecified hypotheses | Also in the referenced statistical analysis plan | P4 |
| **Methods** | | | | |
| Study design | 4 | Present key elements of study design early in the paper | | P10-11 |
| Setting | 5 | Describe the setting, locations, and relevant dates, including periods of recruitment, exposure, follow-up, and data collection | | P10-11 |
| Participants | 6 | (*a*) *Cohort study*—Give the eligibility criteria, and the sources and methods of selection of participants. Describe methods of follow-up<br>*Case-control study*—Give the eligibility criteria, and the sources and methods of case ascertainment and control selection. Give the rationale for the choice of cases and controls<br>*Cross-sectional study*—Give the eligibility criteria, and the sources and methods of selection of participants | | P10-13 |
| | | (*b*) *Cohort study*—For matched studies, give matching criteria and number of exposed and unexposed<br>*Case-control study*—For matched studies, give matching criteria and the number of controls per case | N/A | |
| Variables | 7 | Clearly define all outcomes, exposures, predictors, potential confounders, and effect modifiers. Give diagnostic criteria, if applicable | | P11-12 |
| Data sources/measurement | 8* | For each variable of interest, give sources of data and details of methods of assessment (measurement). Describe comparability of assessment | | P10-11 |

| | | | | |
|---|---|---|---|---|
| | | methods if there is more than one group | | |
| Bias | 9 | Describe any efforts to address potential sources of bias | N/A | |
| Study size | 10 | Explain how the study size was arrived at | | P10-11 and Figure 1 (P27) |
| Quantitative variables | 11 | Explain how quantitative variables were handled in the analyses. If applicable, describe which groupings were chosen and why | | P12 |
| Statistical methods | 12 | (*a*) Describe all statistical methods, including those used to control for confounding | | P12-13 |
| | | (*b*) Describe any methods used to examine subgroups and interactions | | P12-13 |
| | | (*c*) Explain how missing data were addressed | | P12-13 |
| | | (*d*) *Cohort study*—If applicable, explain how loss to follow-up was addressed<br>*Case-control study*—If applicable, explain how matching of cases and controls was addressed<br>*Cross-sectional study*—If applicable, describe analytical methods taking account of sampling strategy | | |
| | | (*e*) Describe any sensitivity analyses | N/A | |

| **Results** | | | | |
|---|---|---|---|---|
| Participants | 13* | (a) Report numbers of individuals at each stage of study—eg numbers potentially eligible, examined for eligibility, confirmed eligible, included in the study, completing follow-up, and analysed | | Table 1 and Figure 1 |
| | | (b) Give reasons for non-participation at each stage | | P8 and Figure 1 |
| | | (c) Consider use of a flow diagram | | Figure 1 |
| Descriptive data | 14* | (a) Give characteristics of study participants (eg demographic, clinical, social) and information on exposures and potential confounders | | Table 1 |
| | | (b) Indicate number of participants with missing data for each variable of interest | N/A | |
| | | (c) *Cohort study*—Summarise follow-up time (eg, average and total amount) | | P5 |
| Outcome data | 15* | *Cohort study*—Report numbers of outcome events or summary measures over time | | Table 1, P7 |
| | | *Case-control study*—Report numbers in each exposure category, or summary measures of exposure | | |
| | | *Cross-sectional study*—Report numbers of outcome events or summary measures | | |

| | | | | |
|---|---|---|---|---|
| Main results | 16 | (*a*) Give unadjusted estimates and, if applicable, confounder-adjusted estimates and their precision (eg, 95% confidence interval). Make clear which confounders were adjusted for and why they were included | | Table 2, 3, 4 |
| | | (*b*) Report category boundaries when continuous variables were categorized | N/A | |
| | | (*c*) If relevant, consider translating estimates of relative risk into absolute risk for a meaningful time period | N/A | |
| Other analyses | 17 | Report other analyses done—eg analyses of subgroups and interactions, and sensitivity analyses | N/A | |
| **Discussion** | | | | |
| Key results | 18 | Summarise key results with reference to study objectives | | P7-10 |
| Limitations | 19 | Discuss limitations of the study, taking into account sources of potential bias or imprecision. Discuss both direction and magnitude of any potential bias | | P7-10 |
| Interpretation | 20 | Give a cautious overall interpretation of results considering objectives, limitations, multiplicity of analyses, results from similar studies, and other relevant evidence | | P7-10 |
| Generalisability | 21 | Discuss the generalisability (external validity) of the study results | | P10 |
| **Other information** | | | | |
| Funding | 22 | Give the source of funding and the role of the funders for the present study and, if applicable, for the original study on which the present article is based | | P14 |

**Supplementary Table 7**

A summary of the models that were used in the analyses. We used two types of models: Generalised Additive Models (GAMs) and Cox Proportional Hazard Models (CPHM). For models A, B and C we also describe any differences with the exposure and covariate/confounding variables when we used the alternative blood donors cohort (as opposed to the nominal primary care cohort). All models were restricted to serology samples that were taken after at least two doses of COVID-19 vaccine, as stated in the methods.

| | Model | | | | | | | |
|---|---|---|---|---|---|---|---|---|
| | A | | B | | C | | D | E |
| Cohort | Primary care | Blood donors | Primary care with at least one risk | Blood donors with at least one risk | Primary care | Blood donors | Primary care | Primary care |
| Model Type | Generalised Additive Model | | | | Cox Proportional Hazard Model | | | |
| Outcome | Tested IgG negative | | | | Tested PCR positive | | COVID-19 hospitalisation or death | |
| Exposure | Number of risks (multimorbidity) | | 24 individual risk categories (comorbidities) | 6 individual risk categories (comorbidities) | Tested IgG negative | | | IgG quantiles |
| Additional covariates/ confounding variables | Age, sex, BMI, vaccine dose & product, advised to shield, care home residency, immuno-suppression status, socio-economic status, time since last vaccine, time since start of pandemic, known prior infection | Age, sex, BMI, vaccine dose & product, advised to shield, socio-economic status, time since last vaccine, time since start of pandemic, known prior infection | Age, sex, BMI, vaccine dose & product, advised to shield, care home residency, immuno-suppression status, socio-economic status, time since last vaccine, time since start of pandemic, known prior infection | Age, sex, BMI, vaccine dose & product, advised to shield, immuno-suppression status, socio-economic status, time since last vaccine, time since start of pandemic, known prior infection | Age, sex, BMI, advised to shield, care home residency, immuno-suppression status, socio-economic status, rural urban classification, received additional vaccines between IgG test and outcome | Age, sex, BMI, advised to shield, immuno-suppression status, socio-economic status, rural urban classification, received additional vaccines between IgG test and outcome | Age, sex, BMI, advised to shield, number of risks, received additional vaccines between IgG test and outcome | Age, sex, BMI, advised to shield, 4 risk groups, number of other risk groups, received additional vaccines between IgG test and outcome |

**Supplementary Table 8**

The percentage of individuals (n=64) in the primary care cohort with certain risk factors, who were in 5+ risk groups and had received at least two doses of any COVID-19 vaccine and tested negative for IgG.

| Risk Group | Percentage of individuals with risk group [%] |
|---|---|
| Coronary Heath Disease | 59.37 |
| Diabetes (Type-II) | 59.37 |
| Chronic Kidney Disease | 54.69 |
| Chronic Obstructive Pulmonary Disease | 42.19 |
| Asthma | 40.63 |
| Severe Mental Health Issues | 40.63 |
| Heart Failure | 35.93 |
| Atrial Fibrillation | 29.69 |
| A prior fracture of hip, wrist, spine or humerus | 29.69 |
| Stroke | 26.56 |

## Supplementary Note 2 – results including single vaccinated individuals

We performed our analyses including individuals whose serum was tested for the presence of IgG when they had only received one dose of any COVID-19 vaccine. It is known that individuals with certain conditions may not mount an immune response after just one dose of the vaccine, therefore we required two doses to have been administered at time of the serology measurement in the main analyses. However, this reduces our sample size significantly as many serology measurements were taken during a point in the pandemic when individuals had only received one dose, therefore it is of interest to also include such samples.

By including an additional 6,287 single-vaccinated individuals we were able to study 23,607 vaccinated individuals. The data showed that of these individuals, 2,633 (11·2%) had at least one negative IgG test result. Of the single-vaccinated individuals, 21·9% of them tested negative for IgG post-first-vaccine, compared with the main result of this paper where 7·3% of double-vaccinated individuals tested negative for IgG.

The adjusted odds of a negative IgG test for those with multimorbidity who were at least double vaccinated at the time of the IgG tests (1·83 [1·29-2·58]) were consistent with all vaccinated individuals (1·94 [1·45-2·60]). This was also consistent for those with certain conditions: haematological cancer (2·27 [1·37-3·76] for those who completed the primary course, and 1·85 [1·21-2·83] when also including single-vaccinated individuals); rare neurological conditions (2·40 [1·30-4·44] compared to 1·94 [1·18-3·19]); respiratory cancer (1·99 [0·85-4·63] compared to 2·32 [1·13-4.78]); and sickle cell disease (1·69 [0·62-4·66] compared to 2·55 [1·16-5·59]).

Risk of SARS-CoV-2 infection was higher amongst those with an insufficient, relative to a sufficient, IgG response (Hazard Ratios [HR] of 1·42 [1·28 - 1·57] and 1·42 [1·16 - 1·73] in the primary care and blood donor cohorts, respectively) when single vaccinated individuals were also included. These results are consistent with our nominal findings obtained for double-vaccinated individuals where the hazard ratios are 1.50 [1.30 - 1.73] and 1.93 [1.42 - 2.64] in the primary care and blood donor cohorts, respectively.

We observed the probability of hospitalisation or death those with an insufficient IgG response to be 3·38 [2·26 - 5·04] in model $D_1$ when we also included the single-vaccinated individuals, this, again, is consistent with the nominal finding of as OR of 3.68 [2.28 - 5.94].

Those who were at least double vaccinated and found with undetectable IgG were a greater risk of hospitalisation or death (HR 9·21 [4·63-18·29]) compared to those with average levels. The risk was similar when including single-vaccinated individuals (HR 8·25 [4·39-15·49]).

## Supplementary Note 3 – sensitivity analyses

We performed sensitivity analyses for models A and B for both the primary care (main manuscript) and blood donors' cohort (Supplementary Note 1). We refer to these as separate models i.e. $A_{PC}$ using the primary care cohort and $A_{BD}$ when using the blood donors' cohort. Differences in these models is details in Table SC3.

In model $A_{PC}$, $B_{PC}$, $A_{BD}$, $B_{BD}$ we used the definition of a negative IgG test result (qualitative result) that is determined by the various assay manufacturers ("nominal" definition) – IgG <33.8 BAU/ml for the primary care cohort and IgG <1.1 arb for the blood donors. We conducted a sensitivity analysis for models A ($A_{PC}$ and $A_{BD}$) [Supplementary Figure 8] and models B ($B_{PC}$ and $B_{BD}$) [Supplementary Figure 9], whereby we used alternative definitions, based on the quantitative IgG measurements, that were either tighter or looser than the assay manufacturers' definition. A loose definition was determined to be IgG < 100 BAU/ml for the primary care measurements and IgG < 5 arb for the blood donor measurements. For the tight definition, we used the lower threshold for which the assays can detect any IgG (<4.8 BAU/ml and <0.1 arb respectively).

For model A, we observed those in increasing number of risk groups to be at greater risk of an undetectable IgG level (tight definition) for both primary care attendees and blood donors. In the primary care cohort ($A_{PC}$), an OR of 5.83[3.23 - 10.53] was observed those in 5+ risk groups using the tight definitions, while the OR was 1.94 [1.35 – 2.77] using the nominal definition of a negative IgG test defined by the measurement assay manufacturer. By contrast, the OR for a negative IgG test results (tight definition) for the blood donors ($A_{BD}$,) was 15.47 [3.31-72.08] compared with 9.23 [2.37-35.87] when using the nominal assay definition.

In model $B_{PC}$, for individuals with haematological cancer we observed an OR of 1.46 [0.90 - 2.36] when using the loose definition of a negative IgG result. By contrast, we observed an OR of 9.67 [5.92 - 15.78] for individuals with haematological cancer, when using a tight definition of a negative IgG test. When using this tight definition we also observed significantly higher ORs of not detecting any IgG after at least two doses of the vaccines if the individual (primary care attendee) was at risk due to: pulmonary hypertension (3.78 [1.51 - 9.50]), rare neurological diseases (3.35 [1.34 - 8.39]), cirrhosis (3.30 [1.71 - 6.38]) a history of heart failure (2.49 [1.49 - 4.16]), coronary heath disease (1.79 [1.25 - 2.57]). For the blood donors ($B_{BD}$), a tight definition of negative IgG (undetectable) yielded an OR of 5.57 [2.18 - 14.24] for blood donors with Type-II diabetes.

### Supplementary Figure 8

Adjusted odds ratios (ORs) with 95% confidence intervals for alternative thresholds defining IgG negativity, for individuals who had received at least two doses of any COVID-19 vaccine at time of serological testing (>14 days after their first vaccine) while attending primary care (left) or donating blood (right).

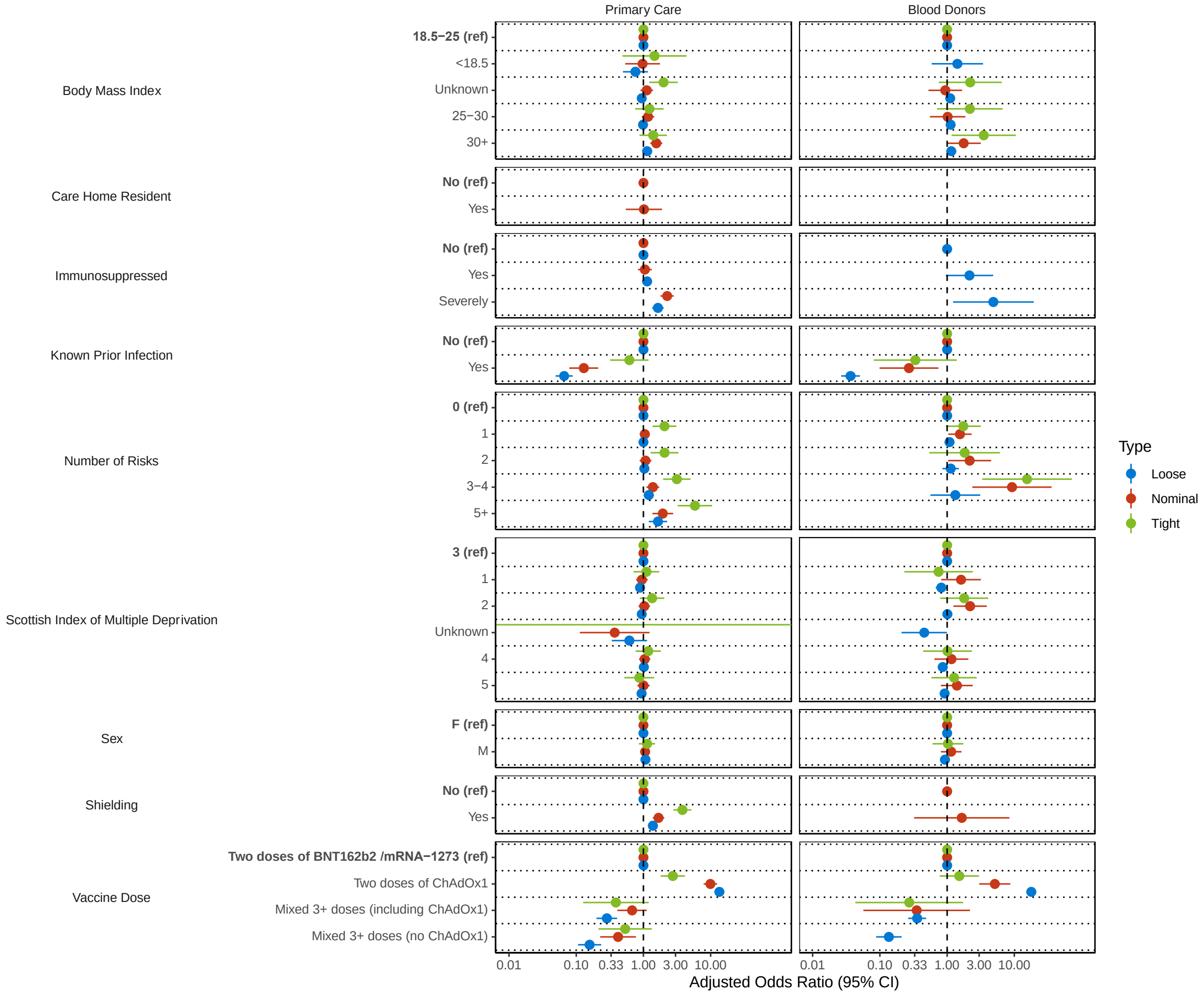


## Supplementary Figure 9

Adjusted odds ratios (ORs) with 95% confidence intervals for alternative thresholds defining IgG negativity, for individuals in at least one QCOVID risk group who had received one dose of any COVID-19 vaccine at time of serological testing (>14 days after their first vaccine) while attending primary care (left) or donating blood (right).

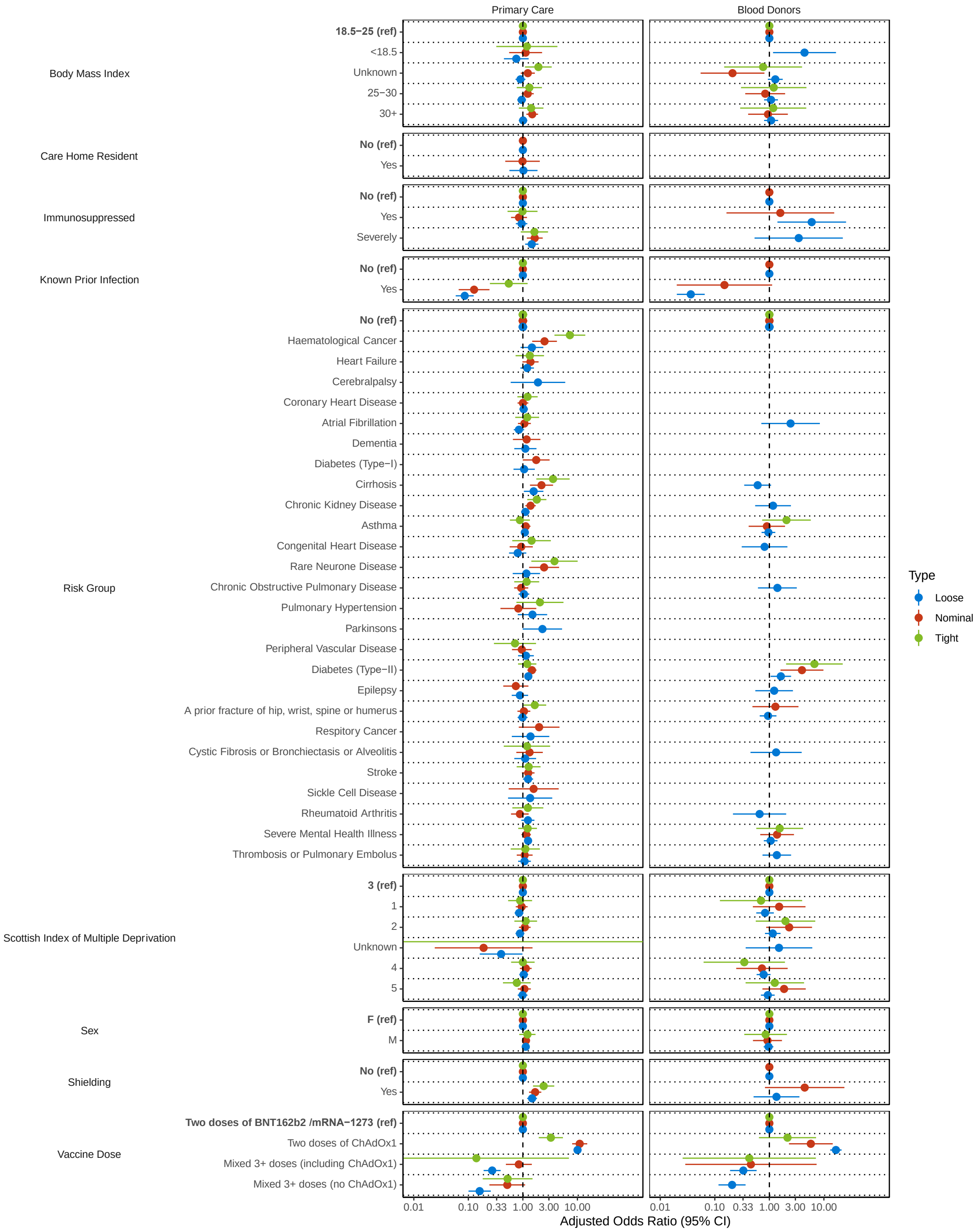
Primary Care
Blood Donors
Body Mass Index
18.5−25 (ref)
<18.5
Unknown
25−30
30+
Care Home Resident
No (ref)
Yes
Immunosuppressed
No (ref)
Yes
Severely
Known Prior Infection
No (ref)
Yes
Risk Group
No (ref)
Haematological Cancer
Heart Failure
Cerebralpalsy
Coronary Heart Disease
Atrial Fibrillation
Dementia
Diabetes (Type−I)
Cirrhosis
Chronic Kidney Disease
Asthma
Congenital Heart Disease
Rare Neurone Disease
Chronic Obstructive Pulmonary Disease
Pulmonary Hypertension
Parkinsons
Peripheral Vascular Disease
Diabetes (Type−II)
Epilepsy
A prior fracture of hip, wrist, spine or humerus
Respitory Cancer
Cystic Fibrosis or Bronchiectasis or Alveolitis
Stroke
Sickle Cell Disease
Rheumatoid Arthritis
Severe Mental Health Illness
Thrombosis or Pulmonary Embolus
Scottish Index of Multiple Deprivation
3 (ref)
1
2
Unknown
4
5
Sex
F (ref)
M
Shielding
No (ref)
Yes
Vaccine Dose
Two doses of BNT162b2 /mRNA−1273 (ref)
Two doses of ChAdOx1
Mixed 3+ doses (including ChAdOx1)
Mixed 3+ doses (no ChAdOx1)
0.01 0.10 0.33 1.00 3.00 10.00
Adjusted Odds Ratio (95% CI)
Type
Loose
Nominal
Tight
